# Changes in Metal-Chelating Metabolites Induced by Drought and a Root Microbiome in Wheat

**DOI:** 10.3390/plants12061209

**Published:** 2023-03-07

**Authors:** Anne J. Anderson, Joshua M. Hortin, Astrid R. Jacobson, David W. Britt, Joan E. McLean

**Affiliations:** 1 Department of Biological Engineering, Utah State University, Logan, UT 84322, USA; 2 Utah Water Research Laboratory, Department of Civil and Environmental Engineering, Utah State University, Logan, UT 84322, USA; 3 Department of Plants, Soils, and Climate, Utah State University, Logan, UT 84322, USA

**Keywords:** amino acids, carboxylic acids, metal complexation, metabolism, microbiome, water stress

## Abstract

The essential metals Cu, Zn, and Fe are involved in many activities required for normal and stress responses in plants and their microbiomes. This paper focuses on how drought and microbial root colonization influence shoot and rhizosphere metabolites with metal-chelation properties. Wheat seedlings, with and without a pseudomonad microbiome, were grown with normal watering or under water-deficit conditions. At harvest, metal-chelating metabolites (amino acids, low molecular weight organic acids (LMWOAs), phenolic acids, and the wheat siderophore) were assessed in shoots and rhizosphere solutions. Shoots accumulated amino acids with drought, but metabolites changed little due to microbial colonization, whereas the active microbiome generally reduced the metabolites in the rhizosphere solutions, a possible factor in the biocontrol of pathogen growth. Geochemical modeling with the rhizosphere metabolites predicted Fe formed Fe–Ca–gluconates, Zn was mainly present as ions, and Cu was chelated with the siderophore 2′-deoxymugineic acid, LMWOAs, and amino acids. Thus, changes in shoot and rhizosphere metabolites caused by drought and microbial root colonization have potential impacts on plant vigor and metal bioavailability.

## 1. Introduction

An increasing problem for agriculture is the loss in growth, yield, and quality due to abiotic stresses such as drought and salinity [1]. These stresses also influence plant responses to microbial pathogens; increased fungal and bacterial diseases occur with abiotic stress [2,3,4]. Plant metabolism changes when the plant becomes stressed. Some of these changes with drought correlate with combating increased reactive oxygen stress, such as increases in activities for peroxidases and superoxide dismutases [5,6]. Other findings for wheat shoots under drought are elevated levels of metabolites that also buffer osmotic stress, including proline, tryptophan, and branched-chain amino acids, but declines in the organic acids [7]. Guo et al. [8] correlate the changes in shoot metabolites with the need to protect cells against osmotic and reactive oxygen stress occurring with drought. However, as discussed below many, of the plant metabolites also are metal chelators and, thus, could be influencing the bioavailability of essential metals.

Cellular metabolism in health and stress is dependent on essential metal elements. Fe, Cu, and Zn are cofactors in enzymes, including catalases, peroxidases, and superoxide dismutases that are vital to stress tolerance, as well as other functions for a growing plant [9]. Indeed beneficial effects of metal-based nanoparticles on plant growth for crops under abiotic stress are reported [10,11], often for plants grown in soils deficient in the essential element supplied by the nanoparticle [12]. These findings suggest that plant performance under stress is suboptimal without balanced metal bioavailability. Plants uptake essential metals through their roots, and the bioavailability of these metals from soil minerals is enhanced in the rhizosphere through chelation by metabolites in the root exudates and products released from microbes in the rhizosphere [13,14,15]. The key metabolites present in the rhizosphere solutions for metal chelation include low molecular weight organic acids, such as citrate and malate, amino acids as well as the specialty products of the plant and microbial siderophores [16].

Analyses of root exudate composition reveal changes with drought [17]. The flux of carbon into wheat roots and their exudates increases with drought leading to greater metabolism from root microbes [18]. Total carbon in root exudates increases with drought for crested wheatgrass, with malate becoming the dominant organic acid [19]. For maize, higher carboxylic acids occur in the root exudates with drought [20]. Changes in phloem transport for soybean under drought stress are proposed to account for altered organic acids and amino acids in root exudates, whereas the sugars exuded by the plant are regulated by metabolism in the root cells [21]. Increases of several osmolytes, including proline and the nonproteogenic amino acid betaine, occur upon rewetting soils growing a wild grass; these increases are in part due to the release of these amino acids from soil microbes where they acted as cell protectants [22].

Drought-imposed changes in rhizosphere metabolites have an impact on microbes colonizing the root [23]. The root exudates from drought-stressed plants stimulate enhanced soil respiration that correlates with increased Gram-negative populations, but not Gram-positive bacteria, in the soil [24]. However, other studies indicate that drought enhances root colonization with Gram-positive bacteria rather than Gram-negative bacteria [25]. Such shifts in the rhizosphere microbial community with drought could influence plant performance. Indeed, stress-related changes in root exudate composition are proposed to feature in the recruitment of a root microbiome, including probiotics that are beneficial for plant health [26,27,28,29,30]. Many of the root exudate metabolites are microbial chemoattractants [31]. For example, citrate and fumarate in root exudates function in chemotaxis and biofilm formation promoting microbial root colonization [32]. Stringlis et al. [33] demonstrate several linked phenomena centering on root exudate composition, the beneficial microbiome, and Fe bioavailability. The growth of *Arabidopsis thaliana* with low Fe nutrition increases the levels of scopoletin in the root exudates. This coumarin, an antimicrobial and an Fe chelator, restricts the growth of two fungal root pathogens but permits root colonization by a beneficial microbe, *Pseudomonas simiae* WCS417. Colonization by *P. simiae* further changes root exudate composition, with increases in citrate, malate, succinate, and phenylalanine. Lombardi et al. [34] find that stress, caused by wounding, salinity or disease, alters tomato root exudates, especially in the levels of oxylipins, to promote growth and attraction of a protective fungus, *Trichoderma harzianum.* Yuan et al. [35] demonstrate that successive growth in the soil of *A. thaliana* challenged with a shoot pathogen changes the composition of the rhizosphere solution to result in the recruitment of a root microbiome that enhances the resistance of the plant to the pathogen. Noted are increases in the rhizosphere solutions of amino acids and long-chain organic acids but reductions in sugars and shorter-chain organic acids. 

In this paper, we focus on how wheat metabolites with roles in metal chelation changed in shoots and the rhizosphere solution imposed by drought. We used a model system involving wheat with and without root colonization by a beneficial bacterium, *Pseudomonas chlororaphis* isolate O6 (*Pc*O6). The wheat used is *Triticum aestivum*, cultivar Juniper, bred for drought tolerance and growth on calcareous soils. The *Pc*O6 strain is from wheat roots grown commercially in calcareous soils under dry land cultivation. Both the plant and this bacterium had survived through periods of heat and drought when isolated at harvest time [36], but whether drought alters the root colonization potential of *Pc*O6 was not known. 

The isolate *Pc*O6 has probiotic effects on plants, including the induction of systemic stress tolerance mechanisms as well as direct antagonism of microbial pathogens. Drought stress relief by root colonization with this pseudomonad involves several mechanisms. A fermentative product from pyruvate, 2R,3R—butanediol, released from root-colonizing *Pc*O6 cells, promotes the closure of stomates, limiting water loss from the plant [37]. Butanediol induces production in the plant cells of the cell signaling molecules nitric oxide and hydrogen peroxide; these molecules can orchestrate the process of stomatal closure [38]. A dense extracellular matrix observed embedding *Pc*O6 cells as they form patchy biofilms on the root surface has over 98% water content contributing to root cell hydration upon drying conditions [39]. Colonization by *Pc*O6 primes for the induction of plant stress-protective genes; genes connected with drought tolerance, such as those required for the synthesis of the osmolyte, trehalose, or encoding dehydrins, are part of the transcript analysis of colonized wheat as discussed by Yang et al. [40]. The cell signaling molecule, galactinol, is another player in the defensive plant responses primed by *Pc*O6 colonization; galactinol applications to cucumber induce pathogenesis-related genes, enhancing resistance to pathogen challenge, drought, and salinity [41]. These traits lead to a strong model system, the host plant, wheat, and a defined bacterium, in which to study changes in shoot and rhizosphere metabolites upon drought stress.

Our analyses of shoot extracts and rhizosphere solutions examined the organic acids and amino acids that chelate the metals Fe, Cu, and Zn, essential for cellular functions in both the plant and its microbial colonists. Sand was used as a solid-defined matrix because it would not contribute organic materials or metals, yet it facilitated the extraction of the rhizosphere solutions [16,39,42,43]. Wheat seedlings were raised with and without an inoculum of *Pc*O6 and with and without a five-day droughting period prior to harvest. The short growth period was to avoid adding fertilizer which would contain ions that potentially change metal chelation. Plant growth and shoot water content were measured to confirm that the drought regime affected plant growth. The colonization of plant roots by *Pc*O6 was assessed at harvest to determine any impact of drought. Rhizosphere solutions were assayed for other generalized properties that could be influencing the system: pH, dissolved organic carbon, and electrical conductivity. The potential chelation patterns for Fe, Cu, and Zn were deduced using geochemical (MINTEQ) modeling based on the values for the amino acid and organic acid levels measured in the rhizosphere solutions. The analyses revealed that changes in metabolites with metal chelation properties differed between the shoot tissues and the rhizosphere when plants were exposed to drought and with root-surface microbial colonization. The geochemical modeling predicted that drought and microbial colonization have differential impacts on the chelation of Fe, Cu, and Zn in the rhizosphere solutions, changes that could affect plant and microbial cell functions.

## 2. Results

### 2.1. Treatment Effects on Seedling Growth, Microbial Colonization, and Rhizosphere Solution Properties

**Figure 1:**
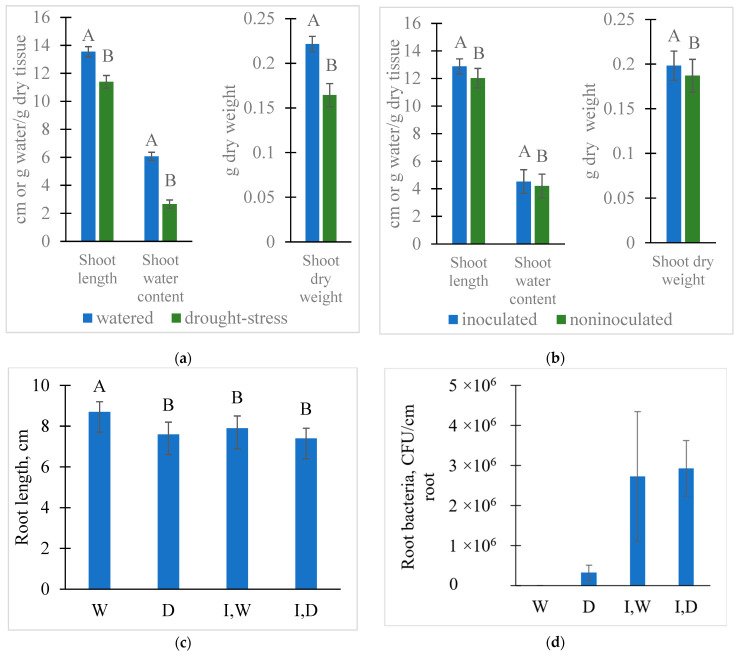
Effects of growth conditions on plant development and root colonization by drought and root colonization by *Pc*O6. Plant were grown with four treatments: W watered, D drought-stressed, I,W inoculated and watered and I,D inoculated and drought stressed. (**a**) Changes in shoot length, shoot dry weight, and water content for watered and drought-stressed plants. (**b**) Changes in shoot length, shoot dry weight, and water content for plants grown without and with inoculation by *Pc*O6. (**c**) Changes in root length induced by drought and inoculation with *Pc*O6. (**d**) The colony forming units (CFU) recovered from the roots of watered and drought-stressed wheat seedlings when grown with or without *Pc*O6 inoculum. Data in (**d**) are shown as CFU/cm of the root. A bar represents the mean from nine independent boxes per treatment, and the 95% confidence intervals are shown. Differing letters show statistically different treatments by Tukey Honestly significant difference test (*p* = 0.05).

The 5 d drought period for the wheat seedlings reduced the growth of the plant, as measured by shoot length and dry mass (Figure 1a, Supplementary Table S1). Drought also reduced the water content of the shoots (Figure 1a). Inoculation with *Pc*O6 lengthened shoots and increased their dry mass but had no effect on shoot water content (Figure 1b). Root length was shortened by drought stress and by colonization with *Pc*O6 (Figure 1c); the roots of *Pc*O6-colonized plants were not further shortened by drought. Roots from drought-stressed seedlings had more intense rhizosheaths when removed from the box than the roots from the watered seedlings. The sand grains in the rhizosheath were retained on the root surface even after extensive rinsing with water. Consequently, the root masses were not reported.

Culturable *Pc*O6 cells were recovered from the inoculated wheat roots independent of whether the plants were watered or under drought stress (Figure 1d). Serial dilution plates from wheat grown with *Pc*O6 inoculum only showed bright orange colonies typical of *Pc*O6, with no evidence of other culturable contaminants (Supplementary Figure S1). The densities of culturable cells of *Pc*O6 recovered from the roots were not affected by drought stress (Figure 1d). Suspensions from the roots of non-inoculated watered plants showed no bacterial colonies on the LB plates. However, white, raised colonies were observed from the suspension of the drought-stressed plants grown without *Pc*O6 inoculum. These were at lower densities than the *Pc*O6 colonies recovered from plants grown with the pseudomonad inoculum (Figure 1d; Supplementary Figure S1).

### 2.2. Metabolite Changes in Shoots with Drought and PcO6 Root Colonization

**Figure 2:**
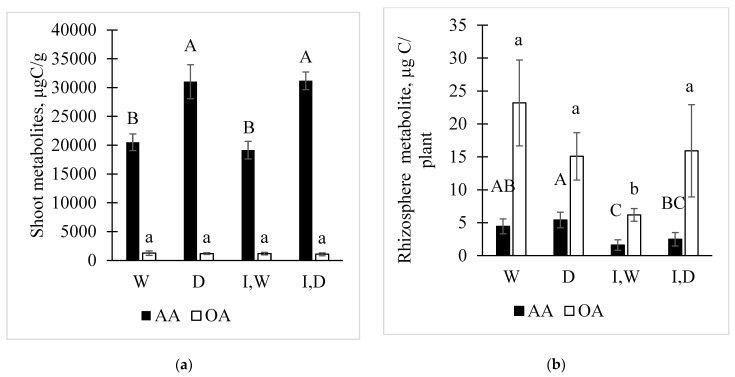
Summed mass of C in amino acids (AA) or organic acids (OA) in (**a**) shoot extracts based on g dry mass shoots or (**b**) the rhizosphere solutions for wheat seedlings grown with four treatments: non-inoculated watered (W) or drought-stressed (D), or *Pc*O6-inoculated watered (I, W) or drought-stressed (I, D). Each bar is the total of the values averaged from nine independent boxes, and error bars are 95% confidence intervals to illustrate the spread of the data. Bars with different lettering are statistically different within each chemical class by Tukey HSD tests (*p* = 0.05). Capital letters cannot be compared to lower-case letters.

Shoot extracts contained an array of proteogenic amino acids, low molecular weight organic acids, and the phenolic acid, ferulic acid, as well as the wheat phytosiderophore, 2′-deoxymugineic acid (DMA) (Supplementary Table S2, Figure 2). The total C mass present in the shoot extracts as amino acids were higher than for the C mass detected as organic acids (Figure 2a). The inverse pattern was seen in the rhizosphere solutions (Figure 2b).

**Figure 3:**
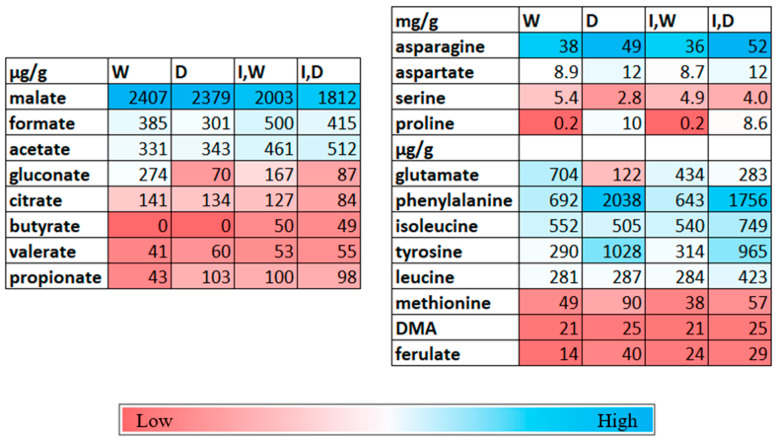
Representation of changes in specific metabolites in shoot extracts after wheat seedling growth under four conditions: non-inoculated watered (W), non-inoculated drought-stressed (D), inoculated with *Pc*O6 (I) and watered (I,W), and inoculated with *Pc*O6 and drought-stressed (I,D). The data shown are µg or mg levels of each metabolite/g dry mass of the shoots based on nine boxes for each treatment. Statistical data are provided in Supplementary Table S2. The heat map shows lower levels in red and higher levels in blue for each metabolite.

For the watered non-inoculated plants, asparagine, as well as aspartate and serine, were at the highest concentrations, mg/g, followed at µg/g concentrations by glutamate, phenylalanine, isoleucine, tyrosine, leucine, and methionine (Figure 3). The tricarboxylic acid (TCA) pathway-related intermediate malate had an mg/g concentration, whereas other acids, formate, acetate, and citrate, were lower at µg/g. A C6-acidic sugar, gluconate, also was present. Analyses of the shoot metabolites did not detect the potential fermentation product, such as lactate, consistent with an aerobic status for leaf cells.

The statistical analysis of the metabolite levels showed complex patterns of change with growth conditions. There was no statistical change with any growth condition for the TCA-related metabolites malate and citrate (Supplementary Table S2). Upon drought stress, acetate did not significantly change. A statistical decrease for formate and gluconate occurred with drought stress (Supplementary Table S2). Drought stress statistically increased the shoot concentrations of several proteogenic amino acids (asparagine, aspartate, proline, leucine, phenylalanine, and tyrosine). Large decreases occurred with glutamate and serine with drought stress (Figure 3, Supplementary Table S2). The increased accumulations to the mg/g level for phenylalanine and tyrosine correlated with higher ferulate levels in the shoot extracts under drought stress. Methionine also increased with drought stress, as did the phytosiderophore, DMA. The most dramatic increase was for the osmolyte proline, from 180 µg/g in the watered plants to about 10,000 µg/g for drought-stressed seedling shoots.

*Pc*O6 colonization caused lesser change than drought stress in shoot metabolites (Figure 3, Supplementary Table S2). Colonization by *Pc*O6 was linked to a lower glutamate level but higher acetate and formate when compared with the extracts from the non-inoculated plants under watered conditions. Other metabolites were not affected. When comparing drought-stressed plants, inoculation resulted in higher serine, isoleucine, and glutamate in shoot extracts. For the organic acids, there were no effects of colonization on malate or citrate, although formate and gluconate increased for the extracts from the drought-stressed shoot extracts from inoculated plants compared with drought-stressed non-inoculated plants. Butyrate was detected in the shoot extracts only from the inoculated plants.

### 2.3. Changes in Metabolites in the Rhizosphere Solutions by Drought and PcO6 Colonization

The pH of the rhizosphere solutions from watered plants was not altered by *Pc*O6 root colonization but was decreased when the plants were exposed to drought from values of pH 7.2 ± 0.1 to pH 6.5 ± 0.1 for both the inoculated and non-inoculated plants (Supplementary Table S3). Although drought had no effect on the measured DOC value, levels decreased by about 40% with bacterial colonization (Supplementary Table S3). However, drought but not microbial colonization increased the electroconductivity of the rhizosphere solution (Supplementary Table S3). Increases in chloride and nitrate ions were seen in the rhizosphere solutions with drought with no effect of *Pc*O6 colonization. Sulfate ions in the rhizosphere solutions were not changed by drought or *Pc*O6 colonization. Shoot concentrations of nitrate but not chloride decreased with drought, but there was no effect of *Pc*O6 root colonization (Supplementary Table S3).

**Figure 4:**
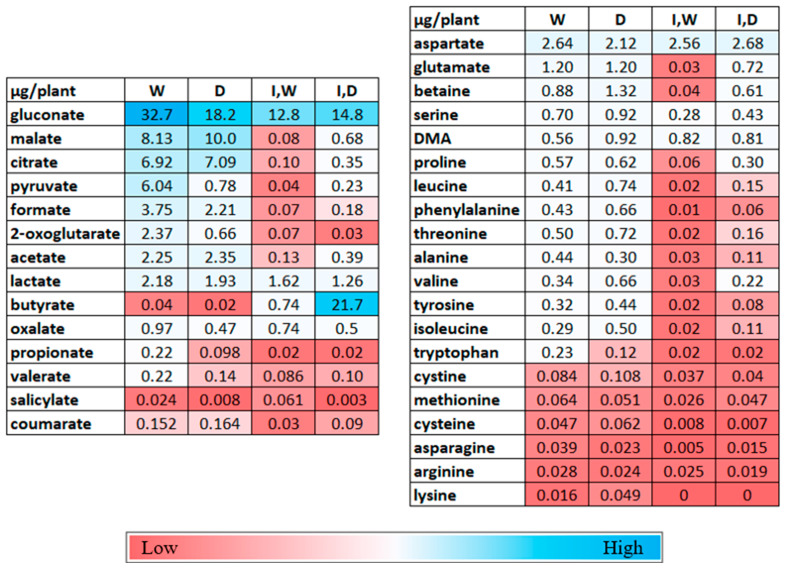
Representation of changes in metabolites in rhizosphere solutions after wheat seedling growth under four conditions: non-inoculated and watered (W), non-inoculated and drought-stressed (D), inoculated with *Pc*O6 (I) with watered growth (I,W), and inoculated with *Pc*O6 and drought-stressed (I,D). The values are mass µg/ plant based on nine boxes for each treatment with statistical analysis as described in Supplementary Table S4.

Metabolite concentrations for 13 organic acids and 20 amino acids in the rhizosphere solutions are shown in Figure 4 and Supplementary Table S4, which shows the statistical analyses. Assessment of C mass in the rhizosphere solutions classified as either organic acids or amino acids is illustrated in Figure 2b. More C was present as organic acids than amino acids, the reverse of the shoot extract. The rhizosphere solutions from the inoculated plants showed the total C mass was little affected by drought, but both the organic acids and amino acids were reduced by *Pc*O6 colonization.

For the watered plants without inoculation, gluconate was the most abundant organic acid in the rhizosphere solutions at 20–25 µg/plant, with the TCA-relevant intermediates (malate, citrate, pyruvate) being between 7–8 and 6 µg/plant followed by formate, about 4 µg/plant, and oxalate and 2-oxoglutarate with about 0.8 µg/plant. For the amino acids, the dominant metabolite, aspartate (about 2.5 µg/plant), was much higher than asparagine 0.04 µg/plant) with glutamate being the next highest at 1.2 µg/plant. The osmolyte, betaine, and proteogenic amino acids serine and threonine were about 0.5 µg/plant and, amino acids phenylalanine, proline, alanine, isoleucine, leucine, tyrosine, and valine being between 0.2 and 0.5 µg/plant. The fermentation acids, lactate, propionate, valerate, and butyrate, also were present, as was the phenolic acid, coumarate. The wheat phytosiderophore, DMA, was detected in the rhizosphere solution, as were two amino acids, proline and betaine, associated with cellular protection against osmotic stress and reactive oxygen species [44,45].

Drought effects on rhizosphere metabolites were subtle. Three metabolites remained statistically unchanged in levels with any growth conditions: aspartate, methionine, and DMA. In non-inoculated seedlings, the same metabolites were dominant in the rhizosphere solutions for the drought-stressed and watered plants. Drought stress resulted in increases in lysine, phenylalanine, and coumarate. Two to four-fold decreases in oxalate propionate, salicylic acid, and the amino acids tryptophan and arginine were observed.

However, *Pc*O6 colonization of plants resulted in large reductions in the concentrations of amino acids and organic acids in the rhizosphere solution (Figure 2 and Figure 4, Supplementary Table S4). These findings agreed with the growth of *Pc*O6 on these substrates as sole nutrient sources using Biolog plates (Supplementary Table S5). The rhizosphere solutions from inoculated plants had lowered asparagine, proline, serine, alanine, cystine, cysteine, tryptophan, arginine, and phenylalanine levels; among the amino acids only lysine, methionine, and aspartate had no statistical change from inoculation. Gluconate, 2-oxoglutarate, lactate, propionate, and coumaric acid decreased with inoculation. Although gluconate decreased with *Pc*O6 colonization, it remained a dominant organic acid.

The drought stress increased levels of several amino and organic acids, specifically under inoculation. These metabolites include betaine, glutamate, leucine, tyrosine, threonine, valine, formate, malate, pyruvate, salicylic acid, and butyrate. The plant growth regulator, salicylic acid, was detected at low levels that decreased with the drought of non-inoculated plants but was elevated to the level from watered plants for the drought-stressed inoculated plants. For the drought-stressed plants colonized with *Pc*O6, the rhizosphere solution was dominated by butyrate and gluconate as organic acids and aspartate as the major amino acid.

### 2.4. Geochemical Modeling of Metal Chelation in the Rhizosphere Solutions

Geochemical modeling predicted that both drought and root colonization affected the potential of the pore waters in the rhizosphere space to hold soluble metals (Table 1). Drought without inoculation of the plant roots was predicted by modeling to increase the loading of the rhizosphere solutions for Cu, whereas Fe concentration was reduced compared to the watered condition (Table 1). Concentrations of Cu and Fe were reduced in the rhizosphere solution by *Pc*O6 colonization of watered plants compared to non-inoculated watered plants. Lower solubility also was predicted for Cu when the colonized plants were watered when compared with the values for the drought-stressed plants. For Fe, solubility in the rhizosphere solution remained lower for all conditions compared to the watered non-inoculated treatment. Soluble Zn in the rhizosphere solutions was relatively unaffected by drought or colonization.

**Figure 5:**
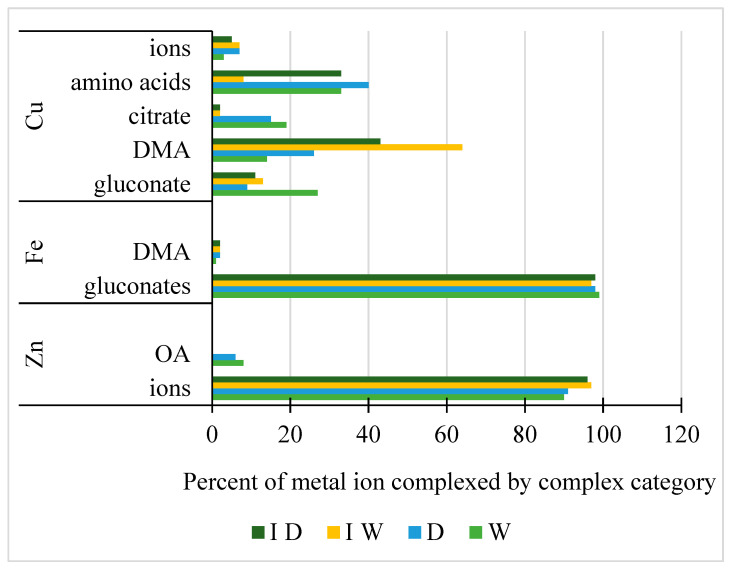
Predicted complexation as % of the total for three essential metals (Cu, Fe, and Zn) when present together in the rhizosphere for four growth conditions: non-inoculated watered (W), non-inoculated (D), inoculated with *Pc*O6 and watered (I W), and inoculated with *Pc*O6 with drought-stress (ID). For modeling pH 7.25 was used. Complexation with amino acids, inorganic anions and organic acids (OA) are shown, as well as chelation with the phytosiderophore, DMA, and the presence of free ions. The full data set is provided in Supplementary data.

The different complexes formed with the three essential metals, Cu, Fe, and Zn, were predicted based on geochemical modeling using the concentrations of metabolites measured in the rhizosphere solutions (Figure 5). Full data sets are provided with details of the conditions in Supplementary Table S6A,B. Free ions as Zn^2+^ were predicted to be present at high amounts, over 90% for the four growth conditions; ZnOH^+^ was a minor form at less than 2% total. Citrate and, to a lesser extent, malate were the dominant organic acid chelators for Zn, with one exception for Zn, gluconate only in the rhizosphere solution from non-inoculated watered plants (Supplementary Table S6B). All Fe was predicted to be complexed as Ca–Fe–gluconates with very minor levels, <1 to 2%, as the DMA complex. However, the predictions for Cu complexation showed complexity. Predictions were for free ion (2–7%), complexation with the mineral carbonate (1–2%), as well as complexation with DMA, organic acids and the amino acids, glutamate, serine, phenylalanine, and valine (Supplementary Table S6A,B).

Drought and *Pc*O6 colonization had little effect on the complexation chemistry of Fe in the rhizosphere solutions because of the dominance of the Ca–Fe–gluconate complexes. Drought also had little effect on Zn complexation, whereas colonization of plants with *Pc*O6 increased free Zn ion levels because the organic acid levels were reduced in the rhizosphere solutions. For Cu, predictions were for drought to decrease the complexation with gluconate but increase the complexation with DMA and amino acids. For the rhizosphere solutions from *Pc*O6-inoculated watered plants, the association of Cu with DMA was dominant and with lesser complexation with amino acids and organic acids. With the drought of the *Pc*O6-colonized plants, predicted changes in the rhizosphere solutions were increased Cu complexation with the amino acids.

## 3. Discussion

The wheat seedlings grown under the described conditions showed anticipated morphological responses to growth with drought; droughting reduced root and shoot length, shoot water content, and shoot dry mass. *Pc*O6 colonizes wheat roots as patchy biofilms with cells embedded in extracellular materials [39], but the period of drought stress in this study did not lessen the recovery of root-associated *Pc*O6 cells. In the non-inoculated plants under drought stress, we suggest that a microbe, initially present as an internal, seed-borne endophyte, became a rhizosphere colonist to account for the white-colored bacteria recovered from these roots. As such, these surface microbes could have contributed exopolymers to the root surface mucilage. Rhizoplane colonization by *Pc*O6, or the uncharacterized endophytes associated with the roots of drought-stressed seedlings, may be involved in the formation of the robust rhizosheaths, where sand grains aggressively coated the root surface. The water-holding capacity of the microbial exopolymers and plant mucilage important in rhizosheath formation is proposed as an adaptive strategy to improve tolerance to drought [46]. Currently, the contributions of root endophytes versus root epiphytic microbes to the metabolites in the rhizosphere solutions are not resolved, but any exopolymer secretions from surface microbes would aid in the retention of moisture around the root. Previous work had shown that a *Bacillus subtilis* endophyte from surface-sterilized wheat seeds, when inoculated onto wheat seeds followed by plant growth, resulted in root-surface colonization by this bacterium [47].

**Figure 6:**
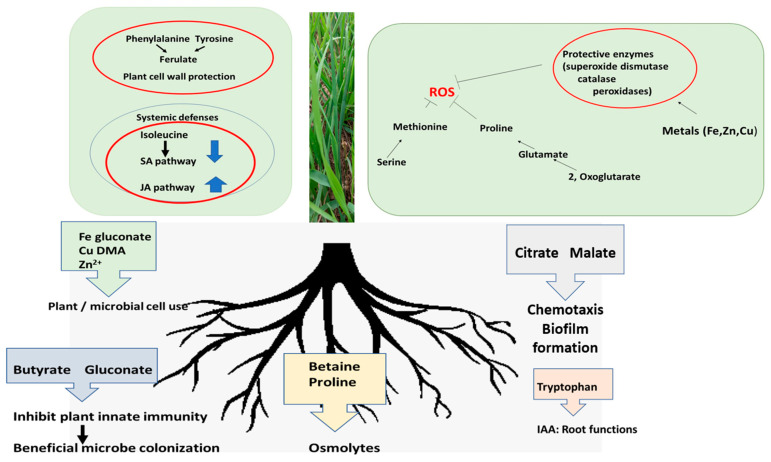
Summary of potential roles of organic acids and amino acids in the shoots and in rhizosphere solutions of wheat seedlings. Many of the metabolites in the shoot cells and enzymes with metal cofactors are related to protection against reactive oxygen stress (ROS). In the rhizosphere solution, the metabolites help nutrition of the plant and microbe, and root structure and promote microbial colonization of the plant root.

The ratios of organic acids and amino acids in the shoot extracts differed from those in the rhizosphere solutions. Shoot extract metabolites were consistent with photosynthetic activity generating TCA and photorespiration intermediates. A proportion of this C-mass would be converted to proteogenic amino acids by the pathways outlined in Supplementary Figure S2A. The presence of nitrate added to the growth matrix as Ca nitrate at planting time ensured an N source for the seedlings and root-colonizing microbes. Potential roles for metabolites that changed with drought are summarized in Figure 6 for the shoot and the rhizosphere solutions.

The observation of increased amino acid concentrations in the shoots due to drought confirmed previous findings in a variety of plants, perhaps due to reduced growth under water limitation, as discussed for wheat by Yadav et al. [48] and Guo et al. [8]. Yadav et al. [48] found the magnitude of increases for several amino acids upon prolonged drought (28 d) allowed the prediction that elevated serine, asparagine, and methionine in the flag leaves of wheat indicated strong drought tolerance of a cultivar. Marček et al. [49] also observed differences in metabolic responses among wheat cultivars with varied drought tolerance. Juniper wheat used for our studies was bred for growth at high altitudes, stress from intense ultraviolet irradiation, without irrigation, and in calcareous soils [50]. The increases in the shoot amino acids, serine, asparagine, and methionine, observed in our studies for the drought-stressed plants were as documented in other studies in drought-tolerant cultivars [48].

In this paper, increases in asparagine, and its precursor, aspartate, occurred with drought in the shoot. One explanation for these increases could be that in the presence of shoot N from abundant nitrate, asparagine, and aspartate formation assisted in maintaining the function of a modified TCA cycle by removal of the C4-intermediate, oxaloacetic acid. During drought, the conversion of citrate to oxaloacetic acid may be shuttled in wheat through an alternative route than the normal TCA cycle generating malate through a bypass involving isocitrate lyase and malate synthase (Supplementary Figure S2A) [51,52]. Indeed, malate had the highest concentration of the TCA intermediates detected in the shoot tissues, and this level was maintained under drought. The bypass avoids enzymes of the TCA cycle that are inactivated by drought-induced reactive oxygen stress. Because 2-oxoglutarate is not produced in the shunt pathway, this alternative pathway could explain the observed lowered glutamate levels in shoot extracts from drought-stressed plants (Supplementary Figure S2B). Lowered glutamate also could be due to its conversion to the protective osmolyte, proline [45], which showed very large accumulations in the drought-stressed shoots. A similar suggestion was made by Guo et al. [8] for drought-stressed wheat. Like our cultivar, when exposed to a five-day drought, enhanced levels of proline were observed by seven days in Australian drought-tolerant cultivars [48]. Proline is correlated with many protective effects for limiting stress in plant cells, including increased osmolarity and oxidative stress [45].

Our observations of decreased shoot serine with drought over the five-day period in wheat seedlings agreed with results from some of the eight Australian wheat cultivars examined after 7 d by Yadav et al. [48] although a switch to higher serine levels occurred by 28 d drought. The stomatal closure induced by drought might explain in part why the serine level was lowered because serine is a transamination product of glycolate generated by photorespiration in the mitochondria [53]. Additionally, in agreement with lowered serine level is the finding of lower formate levels with drought because glycolate can act as a precursor of formate [54]. However, earlier studies with wheat reported that photorespiration increases with drought, at least temporarily, while suggesting that the ROS stress so generated was modified by enhanced peroxidase activity [55]. Another reason for the decrease in serine could be the greater production of methionine during drought, as observed in this paper and by Yadav et al. [48]. Methionine gains its backbone from aspartate but has a methyl group originating from serine. Methionine is important in drought because it is required for the synthesis of glutathione which is involved in reducing ROS stress.

Two other altered metabolites by drought stress in the shoots also may be involved in the protection of the plant, accumulations of acetate and ferulic acid (Supplementary Figure S2A,B). The acetate may be produced *in planta* from pyruvate based on findings by Rasheed et al. [56]. These studies, in Arabidopsis, correlated the higher acetate levels with better drought tolerance through ABA-stimulation of the enzymes that convert pyruvate to acetate [56]. Increased production of ferulic acid upon drought, observed with the wheat shoots, agreed with the changes for triticale reported by Hura et al. [57]. These authors suggested that the ferulic acid could be free or bound to cell wall polysaccharides with functions that provide tolerance to drought, such as lowering light transmission or participation in the mechanisms of reduced leaf growth by limiting cell wall plasticity. The increased levels with the drought of the aromatic amino acids, phenylalanine or tyrosine, could be used in the shikimic pathway and may explain the higher level of the ferulate (Figure S2).

Pseudomonad root colonization had a large impact on the metabolite composition in the rhizosphere solution. The lower concentrations of amino acid and organic acids in the rhizosphere solutions from the colonized plants were consistent with the ability of pseudomonad to catabolize many simple metabolites. Growth of the *Pc*O6 isolate on defined organic acids and amino acids that were detected in the rhizosphere solutions was confirmed by the findings of the Biolog assay, where each biochemical was the sole nutrient source. Limitations of the Biolog assay include that the pseudomonad had each biochemical as the sole nutrient source rather than a mixture as in the rhizosphere and that pseudomonad growth was planktonic rather than as the patchy biofilm seen on the rhizoplane [39]. It is possible that the high aspartate to asparagine ratio observed in the rhizosphere solution could be due to asparaginase activity, a common trait for pseudomonads [58].

Additional important roles for some of the metabolites outside of their participation in house-keeping pathways are highlighted in Figure 6. The gluconate detected in both shoot extracts, and rhizosphere solutions could be produced by the plant or by the plant-associated microbes. There is no characterization of a glucose oxidase in plant cells to convert glucose to gluconate. However, gluconate is a common microbial metabolite. In *Pc*O6, gluconate could be catabolized to C3 intermediates by the Entner–Douderoff pathway. Additionally, in pseudomonads, gluconate is a direct source of ATP because the electrons from the oxidation of gluconate, as well as glucose, may be funneled into the electron transport chain [59]. Gluconate can be excreted by the pseudomonads when other C-sources are being used for growth and then imported or catabolism when those sources are depleted [59]. It is possible that gluconate produced by *Pc*O6, or root endophyte metabolism, is transferred in the xylem to the shoot tissue, thus, explaining its presence in the shoot extracts.

Root colonization with *Pc*O6, in general, lowered the amino acid content of the rhizosphere solutions. For the amino acids, the depletion of tryptophan could relate to its transformation by *Pc*O6 metabolism to IAA, a regulator of root growth and function. We previously demonstrated exogenous tryptophan promoted root thickening and root hair formation consistent with increased IAA production [60]. Maintenance of root hairs would be an important process to ensure water and nutrient uptake during drought [61]. The bacterium also could use arginine to generate polyamines that would stimulate their biofilm formation [62] and drought stress tolerance in the plant [63]. Secretion of betaine in the rhizosphere solution may be a ploy by the plant to maintain the beneficial microbe under drought conditions. The *Pc*O6 genome has genes for betaine transport into the bacterium and followed by its potential catabolism. Other findings indicate that bacteria preferentially import betaine to maintain osmotic balance rather than generate this protectant from its own pathways [64].

Several of the acids observed in the rhizosphere solutions have additional roles connected with plant pathogen resistance. It is thought that root-associated microbes would need to negotiate plant defenses to allow microbial colonization [65]. Yu et al. [66] proposed that gluconate production by probiotic bacteria aids in suppression of innate immunity that otherwise would deter their colonization of the root. Yu et al. [66] suggested that it was the acidification of the rhizosphere by gluconate that was an important feature in the prevention of innate immunity. Thus, a similar role may be suggested for the butyric acid that is found in both the plant tissue and the rhizosphere solutions only when plants are inoculated. Production of butyrate is well documented for fermicutes [67], raising the possibility that *Pc*O6 colonization changes the metabolism of endophytic fermicutes. Butyrate correlates with increased production of gamma aminobutyrate, a general stress metabolite, because it causes plant cell acidification [68]. Butyrate also is linked to the modification of gene expression through the inhibition of histone methylases [69,70]. One enzyme connected with drought tolerance is superoxide dismutase, and butyrate applications enhance this activity in plant tissues [71]. The amino acid isoleucine, which was unchanged with drought in non-inoculated plants but was at higher levels for colonized drought-stressed plants, has other connections with plant defenses. Isoleucine in the plant possibly modulates the cross-talk between the jasmonic acid pathway and the salicylic acid-inducible defense pathways [72,73]. Isoleucine enhances the glycosyl transferase causing glycosylation of salicylic acid to form an inactive complex [72]. However, the complex of isoleucine with jasmonate enhances the activation of JA-dependent plant defenses [74]. Root colonization by *Pc*O6 triggers induced systemic resistance to pathogens through the ethylene/JA-defense pathway rather than the SA pathway [75]. The production of JA also is reported to be essential for wheat to produce the growth regulator, abscisic acid, that regulates mechanisms involved in enhanced drought protection [76].

As discussed, other essential roles for the organic acids and amino acids in the rhizosphere solutions are as metal chelators. In general, the solubility of the three metal oxides is Fe < Cu < Zn based on their solubility products. However, this general order of solubility could be changed by chelation with different metabolites, such as gluconate, to enhance Fe solubility above that of Cu. The predicted complexation of Cu in the rhizosphere was more varied than that of Fe or Zn and illustrated the potential roles of several different metabolites in the rhizosphere solution. Free (uncomplexed) Cu ion concentrations were low because of the strong complexation of Cu with the phytosiderophore DMA, amino acids, primarily glutamate, and the organic acids, citrate and malate. The affinity between Cu and glutamate is interesting in light of the paper by Kim et al. [77], where glutamate is found to be a biostimulant for biological control by an actinomycete promoting its root colonization.

Colonization of roots by *Pc*O6 predicted lower metal solubility in the rhizosphere solutions because of the lower levels of amino acids and organic acids in the rhizosphere solutions. Previous studies [43] found that *Pc*O6 colonization of wheat roots (compared to no inoculation) reduced pore water Cu levels when CuO nanoparticles were in the growth matrix. However, *Pc*O6 does not seem to metabolize DMA [42] (Figure 4), even though it is readily broken down by other soil microbes. This study reaffirmed that Cu-DMA was a dominant complex in the rhizosphere solutions of *Pc*O6-colonized plants with and without drought. It is possible that the complexation of Cu with DMA and the bioavailability of this complex for wheat but not the pseudomonad [42] could be vital for wheat seedling health. In this context, *Pc*O6 was isolated from the root surface of mature wheat grown in calcareous soil where Cu, as well as Zn and Fe, would be limiting due to carbonate concentrations and the alkaline pH.

With Fe, the modeling predicted no free ions and only a small concentration of DMA complexation; rather, Ca–Fe–gluconate chelates were predicted. This finding countered our expectation that Fe would be associated with DMA, which is regarded as the chelating compound for Fe ions in the wheat rhizosphere. Instead, the amount of Fe dissolved depended almost entirely on the concentration of gluconate, and this complexation Fe was essentially independent of root colonization by *Pc*O6, drought or drought of *Pc*O6-colonized plants suggesting this complex was not utilized by *Pc*O6. Gluconate complexation raised possibilities that this speciation could restrict pathogen access to Fe in the rhizosphere and, thus, participate in biological control. Stringlis et al. [33] previously demonstrated the importance of the control of rhizosphere Fe in studies of another root-colonizing pseudomonad where Fe chelation limited pathogen development. The plant bioavailability of these gluconate complexes is unknown. Fe gluconate was less effective for plant loading and growth than chemical chelates for soybean [78], possibly due to less uptake of the complex into the plant [79].

The existence of Zn in the rhizosphere solution predominantly as the free ion is due to its high theoretical solubility and suggested that the normal root uptake channels for the free ion would be functional for Zn loading into the plant [80]. This finding may have importance because Zn is often a limiting factor globally for balanced plant nutrition in soils, a fact that contributes to Zn deficiencies in human diets [81]. Neither drought nor *Pc*O6 colonization was a driving factor for Zn complexation in the rhizosphere solution, but drought did maximize the capacity of the pore water for Zn loading. Studies in maize found lowered water field capacity reduced loading of Cu, Fe, and Zn into the shoot tissues, changes that were mitigated by applications of compost tea which would provide additional organic compounds that could act as Zn chelators to promote bioavailability [82]. Castor bean shoots had lower levels of Cu, Fe, and Zn under drought [83]. Lowered levels of Fe in plants under drought stress could be related to reduced uptake from the roots. Xu et al. [25] showed drought modified the fate of Fe in the sorghum rhizosphere with reduced expression of the Fe-transporter genes. However, drought simultaneously promoted Fe uptake and metabolism in a protective actinomycete that colonized the roots. They concluded that regulation of Fe, through changes in the plant and microbiome, could be a key issue in restricting damage due to ROS during drought.

## 4. Materials and Methods

### 4.1. Wheat Growth with and without a Drought Stress and Colonization by PcO6

The experiment is a 2 × 2 factorial design to evaluate shoot metabolites and root exudates response to the main effects of the presence/absence of PcO6 with and without drought conditions and to evaluate the interactions of these main effects. The method of McManus et al. [16] was followed for the growth of wheat. Briefly, wheat seeds cv. Junipers were surface sterilized with 3% sodium hypochlorite from commercial Clorox for ten min before thorough washing with sterile water. The seeds were placed onto Luria-Bertani (LB) agar to germinate. After 4 d, germinated seeds that showed no signs of bacterial or fungal growth from the seeds on the agar were selected and transferred to Magenta boxes (25 seeds/10 × 7 × 7 cm box) for growth. These boxes contained washed, sterilized white high-purity silica sand (UNIMIC Corp., Boise, ID, USA) (300 g) that was wetted with 45 mL of sterile 3.34 mM Ca nitrate as an electrolyte. Details for the sand preparation and its metal analysis are provided in Supplementary Data Table S7. Other boxes contained sand with 2.5 × 10^5^ colony forming units (CFU)/g *Pc*O6 cells, which had been grown on a chemically defined minimal medium agar lacking added Fe, Cu, or Zn but containing sucrose as the carbon source with magnesium, ammonium, and phosphate ions [84] for 18 h. Cells were obtained by suspension in sterile distilled water from the colonies on the agar surface, pelleted by centrifugation at 10,000× *g* before suspension in sterile Ca nitrate. The 45 mL volume added to each box resulted in wetting to 150% field capacity. Field capacity was determined by allowing the saturated sand to freely drain overnight [16]. The boxes were closed with lids to maintain hydrated status and placed under LED lights (Yescom 225 white LED) (an average of 380 µmol/m^2^/s) with 16 h light/8 h dark cycle. Seedlings were grown in these sealed boxes for 3 d before the drought stress of half of the boxes. Drought stress was imposed by opening the boxes for 2 d to allow evapotranspiration, reducing the water content to 19% field capacity (5.7 ± 1.5 mL/box). These boxes were again closed until harvest maintaining water deficit for the seedlings for a further 3 d. Thus, the seedlings at harvest were 14 d past germination and were grown as watered seedings or with drought stress during the final 5 d of growth. Two independent studies were performed with three and six replicated boxes for the four growth conditions, watered with and without *Pc*O6 inoculation and drought-stressed with and without *Pc*O6 inoculation, for a total number of replicated per treatments of 9 (*n* = 9).

### 4.2. Determination of Plant Growth and Colonization of Plant Roots

At 14 d (10 d after planting and growth with or without 5 d of drought stress before the harvest), the shoot and root tissues were separated, and shoot and root fresh mass and lengths were determined. The water content (g water/g dry tissue) of the shoots was determined by drying the tissues at 60 °C and calculating water loss for each shoot sample with the leaves pooled by treatment. A subsample of roots from the boxes across independent studies (*n* = 1–2 boxes per study) was assayed for colonization by *Pc*O6 and/or endophytes by transfer of a section of the intact root to 10 mL sterile water. For *Pc*O6-inoculated plants, three measurements using three roots per box were made, whereas non-inoculated boxes had one measurement with one root per box. After vortexing for one min to release bacterial cells from the root surfaces ten-fold serial dilutions were made and, 10 µL triplicate samples were plated onto LB medium for growth at 25 °C. Colonies were counted, and their appearance was noted. Colonies of *Pc*O6 were identified by their characteristic orange coloration. Colony counts were normalized to sampled root length.

### 4.3. Shoot Extraction and Analysis

At harvest, three leaves were selected from the shoots and immediately frozen at −70 °C. Tissue was lyophilized before being ground three times in 2 mL centrifuge tubes with stainless steel beads using a Geno/Grinder set at 1000 strokes/min for 60 s. The sample holders were equilibrated in liquid nitrogen-cooled blocks to ensure the samples remained cold. The samples were extracted by methods based on those of Zorb et al. [85]. Briefly, ~3 mg dry tissue was weighed into a 2 mL centrifuge tube with Lysing Matrix G (MP Biomedicals) containing 1.6 mm silicon carbide and 2 mm glass beads, and the exact amount of dry tissue was recorded for normalization. One mL of 80% methanol was added, and the tubes were shaken three times at 6.5 m/s for 45 s with 5 min cooldown time between runs. The samples were centrifuged at 21,000 × *g* for 10 min, and the supernatants were diluted 1:5 fold and analyzed for organic acids and amino acids.

Organic acids were quantified by ion chromatography (Dionex method 123) with a Dionex ICS-3000 (ThermoFisher Scientific, Waltham, MA, USA) against external standards. Amino acids were analyzed by liquid chromatography triple quadrupole mass spectrometry (LC-QqQ-MS) (Agilent 1290 LC coupled with Agilent 6490 QqQ-MS) with an Imtakt amino acid column (50 × 3 mm) using Imtakt method T1794E [86] with mobile phases of 100 mM ammonium formate and acetonitrile with 0.1% formic acid. Analytes were quantified using C^13^- and N^15^-mass-labeled amino acids as internal standards (Sigma—Aldrich 767964, St Louis, MO, USA, [87]). Details of the methods, with the full list of analytes, their transitions, and retention times, are shown in Supplementary Table S8A–E. The organic acids and amino acids detected with confidence are listed in Supplementary Table S2 for the shoots. The wheat siderophore, 2′-deoxymugineic acid (DMA), as well as phenolic acids, were quantified by LC-QqQ-MS with an Agilent Eclipse C18 column (2.1 × 50 mm) [88] with 20 mM ammonium formate with 0.1% formic acid and acetonitrile with 0.1% formic acid mobile phases. Spectra for the ions obtained from the DMA standard and DMA in the plant extracts are provided in Supplementary Figure S3. Blanks, blank method spikes, and matrix spikes were analyzed to ensure quality extractions. Quality control data for select metabolites from the shoot extracts are discussed. Supplementary Figure S4 shows the values for metabolites with consistent but lower-than-expected recoveries. Those metabolites with inconsistent recoveries were not included in the results.

### 4.4. Extraction of Rhizosphere Solutions and Assay of Properties

Plants were harvested 10 d after the germinated seedlings were transferred to the sand matrix. Growth was without drought stress for five d and with/ without drought stress for the five days prior to harvest. Rhizosphere solutions were obtained by transfer of the sand removed from the boxes without the root mass to sterile, acid-rinsed funnels stoppered with glass wool. A vacuum was used to remove the aqueous phase containing the root exudates. The watered treatments produced 11 ± 1 mL of filtrates per box. The sand from the drought treatments was amended with 25 mL of water prior to extraction to allow recovery of the rhizosphere solutions. After this addition, the sand from the boxes with the drought treatments generated 9.5 ± 0.5 mL per box. The exudates were immediately filtered through 0.2 µm nylon filters. Aliquots for trace elements analysis were acidified with 1% nitric acid. The metabolites were assayed by the same methods provided in the previous section, Section 4.3, and those metabolites with robust identification are identified in Supplementary Table S4.

The water content of sand at harvest was measured gravimetrically by a subsample after thoroughly mixing the sand and drying overnight at 105 °C. Additionally, the boxes with drought stress grown under the same conditions but not used for root exudate extraction were prepared to measure sand water content without adding 25 mL of water at harvest. The final sand water contents for the watered plants were 110 ± 10% field capacity, whereas the drought boxes were at 19 ± 5% field capacity.

The pH, electrical conductivity (EC), dissolved organic carbon (DOC), and metabolites in the rhizosphere solutions were analyzed immediately. The pH and EC were measured by standard methods [89]. The DOC was measured by combustion [89] after 1:10 dilution with an Apollo 9000 carbon analyzer (Teledyne Tekmar, Mason, OH, USA). Metals were quantified by using an Agilent 7700x inductively coupled mass spectrometer using EPA Method 6020 [90].

### 4.5. Data Transformations, Statistical Analysis, and QC

All measurements were subject to standard quality control as specified by the USEPA [90]: after calibration, instrument blanks and calibration verification samples were run every 10 samples to verify no contamination and continued accurate quantitation of the analytes. Matrix spike duplicates were performed on 10 percent of the samples to determine the precision of the analysis and ensure full recovery of the analytes within the sample matrix.

All rhizosphere solution data are expressed on a mass basis per plant (mg or µg per plant), achieved by multiplying measured analyte concentration (mg/L or µg/L) by the volume of solution in each box at harvesting and dividing by the number of plants. The purpose of this transformation was to express all analytes on a common basis for each of the four growth conditions due to the differing water levels in the watered/drought treatments. Normalization of the exudates to root mass was not possible for two main reasons: the inability to successfully remove interfering sand grains without destruction of the root tissues and the fact that some metabolites originate from bacteria, so normalizing the exudates to roots is suspect. The limitation of this approach is that root mass influences exudate production. However, other processes in the rhizosphere, such as metabolism by rhizosphere bacteria, already obscure the root mass–exudate relationship.

All shoot metabolite data are reported on a shoot dry weight basis (mass metabolite/g dry weight). When summed, the concentrations of the metabolites regarded as either amino acids or organic acids are first normalized to the amount of C in each individual metabolite as a fraction of its molecular weight. After summation, these values were normalized to the treatment (for rhizosphere metabolites, µg C) or the dry weight of the shoot (µg C/g or mg C/g).

The JMP 8 (SAS Institute, Cary, NC, USA) computer program was used for all statistical analyses. Treatment outliers were identified by the robust fit outlier test, and a maximum of one outlier per treatment was discarded. Data that were censored from the method detection limit were imputed using a log-normal distribution of valid data and assigned a value randomly based on the extrapolation of the log-normal distribution [91]. Statistical differences were determined by Student’s *t*-test or two-way ANOVA at α = 0.05 when significant, followed by the Tukey honestly significant difference (Tukey HSD) test. Responses were blocked by trial after best fit Box Cox transformation to satisfy the equal variance and normal distribution requirements. When variances were still unequal, a Welch’s *t*-test did not reveal changes in the test outcome.

### 4.6. Geochemical Modeling

The intent of geochemical modeling (Visual MINTEQ Ver. 3.1 [92]) was to determine how the modifications of the rhizosphere metabolites by drought and/or *Pc*O6 impacted the metal chelation potential of the rhizosphere solutions. The model inputs for each treatment were the concentration of each of the rhizosphere metabolites detected at harvest, along with major cations and anions. The model input for the atmosphere was the normal 400 ppm CO_2_. Because all components were detected in the soluble state in the rhizosphere solutions, the model was run without allowing the precipitation of complexes.

Equilibrium constants for metal–DMA and metal–gluconate complexes were added to the standard MINTEQ database as previously described by McManus et al. [16], except Fe–gluconate complexes with 2:1 ligand: metal structures were excluded for causing errors in the program. A solid phase containing crystalline tenorite (CuO), zincite (ZnO), and goethite (FeO(OH)) was added to the program. The metal oxides were chosen because our analyses of calcareous soils (where pH limits metal solubility) have indicated that these metals would be in the amorphous and crystalline oxide phases. The pH was fixed at 7.25 (average of both watered treatments) because the pH of the drought-stress treatments could have been influenced by the need to adjust volumes of pore waters at harvest; pH has a large impact on metal oxide solubility increasing with acidity. Since the labile exchangeable metals can vary among soils and are likely to be bioavailable regardless of exudates, a relatively insoluble mineral phase provided a good test of the less labile metals.

## 5. Conclusions

In summary, this metabolite study indicated that a short drought period influenced shifts in amino acid concentrations more than the organic acids in the shoots of wheat seedlings. In contrast, changes in the rhizosphere solutions due to drought for the metabolites studied were minimal compared to those induced by microbial colonization. The consequences of these changes are highlighted in the summary Figure 6. The reduction in organic acids and amino acids in the rhizosphere with *Pc*O6 colonization of the wheat roots could be important in biological control by limiting an array of readily available substrates and bioavailable Fe for pathogen growth. An exception to decreases in metabolites accompanying root colonization was increased levels of butyrate in both shoots and rhizosphere solutions for the *Pc*O6-colonized plants. Shoot tissues showed anticipated increases in the protectant amino acid proline with drought. However, both proline and betaine were depleted in rhizosphere solutions of plants with pseudomonad colonization consistent with microbial use of these protectant amino acids. Geochemical modeling using the concentrations of the metabolites in the rhizosphere solutions shows different chelation patterns that influenced the extent of free ions for Cu, Fe, and Zn. These findings suggested that metal bioavailability for the plant might be altered by drought and pseudomonad colonization.

## Figures and Tables

**Table 1:** Effect of changes in metabolites in rhizosphere solutions on the concentrations of soluble Cu, Fe, or Zn. The predictions are based on the concentrations of the metabolites detected in the rhizosphere solutions obtained from plants grown with and without drought and with and without inoculation by *Pc*O6. Dissolutions are normalized by setting the value of the watered, non-inoculated treatment to 100%. The molar levels of the three soluble metals in the watered, non-inoculated treatment (defined as 100%) are Cu: 5.29 × 10^−6^ M (336 µg/L), Zn: 8.13 × 10^−4^ M (53.2 mg/L), and Fe: 1.4 × 10^−4^ M (7.8 mg/L).

Growth Condition	Normalized % Cu	Normalized % Zn	Normalized % Fe
Non-inoculated, watered	100	100	100
Non-inoculated, drought-stressed	137	105	56
*Pc*O6-colonized, watered	54	91	39
*Pc*O6-colonized, drought-stressed	77	98	45

## Data Availability

The data presented in this study are available in this article and the Supplementary Materials.

## Supplementary Materials

The following supporting information can be downloaded at: https://www.mdpi.com/article/10.3390/plants12061209/s1, Table S1: Growth parameters of plants; Table S2: Shoot extract metabolites with statistics; Table S3: Chemical properties of the rhizosphere solutions and shoot extracts; Table S4: Rhizosphere solution metabolites with statistics; Table S5: Representation of growth of *Pc*O6 with designates substrates as sole carbon sources; Table S6: Results of geochemical modeling; Table S7: Essential metal detection in washed and muffle-furnace treated sand; Table S8: Details of metabolite analyses; Figure S1: Microbial colony growth from root washes; Figure S2: Anticipated major metabolic pathways in wheat; Figure S3: Quality control studies for shoot extracts. References [93,94] are in the Supplementary Materials.
