# Supplementary material for: Changes in Metal-Chelating Metabolites Induced by Drought and a Root Microbiome in Wheat
Source: Plants (Basel). 2023 Mar 7;12(6):1209. doi: 10.3390/plants12061209 (PMC10055107; doi:10.3390/plants12061209)
Supplement: Supplementary file 1 [file plants-12-01209-s001.zip › plants-2176607-supplementary.pdf]

Supplemental Information

Supplemental Table S1. Growth parameters of plants grown with water or drought – stress, with and without *Pc*O6 inoculation.

	No inoculation		*Pc*O6 inoculation	
	Water (n = 9)	Drought (n = 9)	Water (n = 9)	Drought (n = 9)
Shoot length (cm)	13.2 ± 0.6	10.9 ± 0.7	13.9 ± 0.3	11.8 ± 0.4
Root length (cm)	8.7 ± 0.5	7.6 ± 0.6	7.9 ± 0.6	7.4 ± 0.5
Shoot water content (g/g dry weight)	5.96 ± 0.34	2.46 ± 0.21	6.18 ± 0.48	2.88 ± 0.53
Shoot dry mass (mg)	221 ± 11	153 ± 14	222 ± 14	174 ± 20

Supplemental Table S2. Shoot metabolites with statistical analyses.

Shoot amino acids with main effects only				
	W	D	I, W	I, D
asparagine mg/g	37.4 ± 2.6 B	50.4 ± 4.1A	43.7 ± 3.2	
aspartic acid mg/g	8.80 ± 0.70 B	11.6 ± 0.88 A	10.2 ± 0.72	
proline mg/g	0.181 ± 0.01 B	9.57 ± 1.9 A	4.88 ± 1.85	
DMA µg/g	20.7 ± 1.1 B	24.8 ± 1.9 A	22.7 ± 1.3	
leucine µg/g	282 ± 36 B	355 ± 82 A	284 ±36 B	354 ± 82 A
methionine µg/g	43.5 ± 9.5 B	73.5 ± 19 A	69.5 ± 20 A	47.5 ± 11 B
phenylalanine µg/g	667 ± 83 B	1897 ± 257 A	1282 ± 247	
tyrosine µg/g	302 ± 43 B	996 ± 104 A	649 ± 132	

Shoot amino acids and phenolic acid with interactions				
	No inoculation		*Pc*O6 inoculated	
	Water (n = 9)	Drought (n = 9)	Water (n = 9)	Drought (n = 9)
ferulic µg/g	14.3 ± 2.0 BC	39.7 ± 9.6 A	24.4 ± 5.5 C	29.2 ± 5.2 AB
glutamate µg/g	704 ± 99 A	122 ± 40 C	434 ± 96 B	283 ± 115 BC
isoleucine µg/g	552 ± 89 B	505 ± 87 B	540 ± 55 AB	749 ± 151 A
serine mg/g	5.43 ± 0.53 A	2.75 ± 0.31 C	4.92 ± 0.27 A	3.97 ± 1.24 B

Shoot organic acids with main effects only				
	Water	Drought	No *Pc*O6	*Pc*O6
gluconate µg/g	220 ± 65 A	78.9 ± 18 B	150 ± 40	
acetate µg/g	412 ± 41		337 ± 39 B	486 ± 56 A
formate µg/g	443 ± 51 A	358 ± 44 B	343 ± 34 B	458 ± 52 A
malate µg/g	2150 ± 1240			
citrate µg/g	122 ± 32			
lactate µg/g	<MDL			
propionate µg/g	<MDL			
butyrate µg/g	<MDL		66.4 ± 86 A	49.4 ± 16 B

Averages with ± 95% confidence intervals are shown in Table S2 for amino acids and organic acids in shoot extracts based on g dry weight of shoots. Different letters are statistically different by Tukey HSD tests. < MDL is less than the method detection limit.

Supplemental Table S3. Chemical properties of the rhizosphere solutions and shoot extracts for plants grown with water or drought and with and without *Pc*O6 inoculation. Values are average ± 95% confidence interval.

Rhizosphere solutions				
	No inoculation		*Pc*O6 inoculation	
	Water (n = 9)	Drought (n = 9)	Water (n = 9)	Drought (n = 9)
pH	7.19 ± 0.11	6.43 ± 0.14	7.25 ± 0.12	6.65 ± 0.14
EC (µS/cm)	278 ± 15	493 ± 31	265 ± 31	456 ± 61
DOC (mg C per plant)	0.33±0.0.10	0.32±0.03	0.19±0.04	0.23±0.05
Cl (µg per plant)	2.03±0.34	3.33±0.43	1.77±0.39	2.66±0.30
NO_3-N (µg per plant)	28.0±6.1	55.4±14	20.9±4.6	55.5±10
SO_4 (µg per plant)	1.32±0.54	2.35±0.49	1.26±0.32	1.96±0.22
Shoot extracts				
	No inoculation		*Pc*O6 inoculation	
µg/g	Water (n = 9)	Drought (n = 9)	Water (n = 9)	Drought (n = 9)

Chloride	2380 ± 280	2430 ± 220	2340 ± 290	2440 ± 290
Nitrate	4120 ± 520	2490 ± 150	4040 ± 580	2650 ± 250

Supplemental Table S4. Rhizosphere solution metabolites with statistical analyses.

**Rhizosphere solution organic acids with main effects only**				
µg per plant	Water	Drought	No *Pc*O6	*Pc*O6
Gluconate	19.6±4.2		25.9±7.4 A	13.8±2.4 B
Oxalate	0.85±0.2 A	0.48±0.06 B	0.67±0.10	
2-Oxoglutarate	0.80±0.57		1.61±1.1 A	0.03±0.02 B
Lactate	1.75±0.28		2.06±0.44 A	1.44±0.32 B
Propionate	0.13±0.09 A	0.062±0.03 B	0.16±0.08 A	0.02±0.01 B
Isovalerate	<MDL			
Salicylic acid	0.043±0.04 A	0.0056±0.002 B	0.016±0.006 B	0.032±0.04 A
Coumaric acid	0.091±0.04 B	0.13±0.05 A	0.16±0.04 A	0.06±0.04 B

**Rhizosphere solution organic acids with interactions**				
	No inoculation		*Pc*O6 inoculated	
µg per plant	Water (n = 9)	Drought (n = 9)	Water (n = 9)	Drought (n = 9)
Acetate	2.25±0.48 A	2.34±1.89 A	0.126±0.06 B	0.39±0.31 B
Formate	3.75±0.87 A	2.20±0.33 B	0.071±0.03 D	0.18±0.05 C
Malate	8.12±2.8 A	10.0±3.4 A	0.081±0.027 C	0.68±0.31 B
Citrate	6.92±3.48 A	7.09±3.68 A	0.101±0.05 B	0.35±0.22 B

Pyruvate	6.04±4.1 A	0.78±0.34 A	0.039±0.018 C	0.23±0.07 B
Valerate	0.22±0.06 A	0.14±0.03 AB	0.086±0.02 B	0.102±0.03 B
Butyrate	0.04±0.03 BC	0.02±0.02 C	0.74±0.61 B	21.7±10.1 A
**Rhizosphere solution amino acids with main effects only**				
µg per plant	Water	Drought	No *Pc*O6	*Pc*O6
Asparagine	0.021±0.008		0.031±0.0121 A	0.0096±0.004 B
Aspartate	2.49±0.66			
Arginine	0.026±0.005 A	0.022±0.004 B	0.027±0.004 A	0.022±0.006 B
Phenylalanine	0.22 ± 0.14 B	0.38 ± 0.18 A	0.55 ± 0.14 A	0.036 ± 0.02 B
Methionine	0.047±0.01			
Proline	0.38±0.15		0.59±0.23 A	0.18±0.14 B
Serine	0.58±0.16		0.81±0.29 A	0.36±0.10 B
Alanine	0.22 ± 0.13		0.37 ±0.24 A	0.067±0.05 B
Cystine	0.067±0.021		0.096±0.035 A	0.039±0.017 B
Tryptophan	0.13±0.07 A	0.072±0.03 B	0.18±0.06 A	0.02±0.005 B
Cysteine	0.031 ±0.016		0.054±0.029	0.007±0.002
Histidine	<MDL			
Lysine	0.016±0.005 B	0.047±0.018 A	0.032±0.012	
DMA	0.78±0.21			

**Rhizosphere solution amino acids with interactions**				
	No inoculation		*Pc*O6 inoculation	
µg per plant	Water (n = 9)	Drought (n = 9)	Water (n = 9)	Drought (n = 9)
Betaine	0.88±0.20 AB	1.33±0.40 A	0.039±0.021 C	0.61±0.28 B
Glutamate	1.21±0.55 A	1.20±0.51 A	0.027±0.009 B	0.73±0.28 A
Isoleucine	0.28±0.17 A	0.50±0.24 A	0.019±0.005 B	0.11±0.031 B
Leucine	0.41±0.22 AB	0.74±0.32 A	0.016±0.007 C	0.15±0.07 B
Tyrosine	0.32±0.13 A	0.44±0.10 A	0.019±0.006 C	0.084±0.042 B
Threonine	0.50±0.26 A	0.72±0.28 A	0.024±0.015 C	0.16±0.10 B
Valine	0.34±0.18 A	0.66±0.36 A	0.033±0.011 B	0.22±0.07 A

The values shown are the averages with ± 95% confidence intervals for the specific organic acids and amino acids detected in the rhizosphere solution, µg per plant. Different letters are statistically different by Tukey HSD tests. < MDL is less than the method detection limit.

Supplemental Table S5. Representation of growth of *Pc*O6 with designated substrates as sole carbon sources as determined at stationary - phase in Biolog plates. The size of the bar indicates relative growth of the cells in liquid shake culture as determined by chromogen accumulation dependent on active pseudomonad metabolism. Blue bars show growth with sugars and organic acids. Green bars show growth on L amino acids and metabolites.

**trehalose**	71		**aspartate**	85
**sucrose**	54		**asparagine**	88
**glucose**	95		**glutamate**	72
**fructose**	7		**glutamine**	128
**gluconate**	63		**alanine**	150
**lactate**	241		**serine**	156
**formate**	202		**glycine**	65
**butyrate**	80		**valine**	113
**GABA**	127		**arginine**	71
**pyruvate**	22		**isoleucine**	17
**citrate**	100		**putrescine**	70
**malate**	83		**proline**	38

Supplemental Tables S6. Results of geochemical modeling for the complexes predicted to be present for Zn, Fe and Cu in the rhizosphere solutions.

The data shown in Supplemental Tables S6A were used to prepare Fig. 5 in the text of the paper for predicted complexation by % determined by geochemical modeling. Plants were grown with normal watering (W) or with drought (D) with (I) or without inoculation. The data in Table S6B set show more details of the predicted complexes by % for the metals when present together in the rhizosphere solution.

Table S6A

		%,W	%, D	%, IW	%, I D
Zn	ions	90	91	97	96
	organic acids	8	6	0.1	0.2
Fe	gluconates	99	98	97	98
	DMA	1	2	2	2
Cu	gluconate	27	9	13	11
	DMA	14	26	64	43
	citrate	19	15	2	2
	amino acids	33	40	8	33
	ions	3	7	7	5
	carbonate	1	1	2	1

Table S6B

	%, W	%, D	%, I W	%, I D
Zn+2	88.674	89.763	95.196	94.53
ZnOH+	1.254	1.217	1.374	1.301
Zn-Citrate-	3.301	2.864	0.086	0.17
Zn-Malate (aq)	2.342	2.547	0.027	0.194
ZnGluconate	1.4	0	0	0
CaFeGluconate(OH)3	82.335	81.521	80.727	81.297
CaFeGluconate(OH)4	16.852	16.392	16.64	16.44
Fe-DMA-OH	0.573	1.907	2.373	2.065
Cu2Gluconate2(OH)3	27	8	13	11
Cu-Citrate-	19.327	14.75	1.587	1.741
Cu-Glutamate (aq)	18	19	1.655	22
Cu-DMA	14.441	25.679	63.279	43.374
Cu-2Ser	6.705	11.306	4.413	6.434
Cu-Phe+	3.218	7	0.345	1.033
Cu-Val+	3.178	8	0.941	4.156
Cu+2	1.677	1.943	4.873	3.67
Cu-Malate (aq)	1.239	1.45	0.04	0.202
Cu-Oxalate (aq)	1.131	0.383	5	1.459
CuOH+	0.774	0.856	2.306	1.644
CuCO3 (aq)	0.675	0.734	2.026	1.417

Supplemental Table S7. Essential metal detection in washed and muffle- furnace treated sand.

	Fe<br>mg /kg dry sand	Zn<br>mg /kg dry sand	Cu<br>mg /kg dry sand
Water soluble (2:1 DI water sand)	1.4	0.01	0.01
Nitric acid digestion (EPA method 3050)	347	2.2	0.4
DTPA-ABC extraction	2.8	0.1	0.3
Nutrient level considered low for plant fertility by DTPA extraction	<3	<1	<0.2

White high purity silica sand (UNIMIN Corp., ID, US), with particle size such that 75 % was retained on a 40-mesh sieve, was washed by deionized water (DI) three times, before heating overnight in a 550 °C muffle furnace to remove organic matter. The muffled sand was washed in distilled deionized water, and oven dried at 150 °C before analyses for metal content and being used for plant growth. The metal contents for Cu, Fe and Zn in the prepared sand were lower than the minimal levels proposed for plant fertility according to James and Topper (2010).

Supplemental Table S8A-E. Details of metabolite analyses

The QQQ method uses the Intrada Amino Acid normal phase chromatographic column to separate and determine amino acids. A second C18 column is used to separate and determine phenolic acids and DMA. Instrument operation conditions are shown in Table 8A:

Table S8A. HPLC and MS conditions.

**HPLC Conditions**		
Column	Intrada	C18
	2.1 × 50 mm, 3 µm,	2.1 × 50 mm, 1.8 µm,
Mobile phase A	100 mM ammonium formate in water	20 mM ammonium formate in water at pH = 3
Mobile phase B	ACN with 0.1 FA	20 mM aqueous ammonium formate at pH = 3 in 9:1 ACN/water
Flow rate	0.60 mL/min	0.20 mL/min
Column temperature	35 °C	35 °C
Injection volume	2 µL	2 µL
Total run time	12 minutes	10 minutes
Gradient<br>Time (min) %B	0 85	0 2
	3 85	1 2
	10 0	8 50
	12 85	10 2

**MS Conditions**	
Ionization mode	ESI Positive/Negative
Gas temperature	330 °C
Gas flow	13.0 L/min
Nebulizer	35 psi
Sheath gas temperature	390 °C
Sheath gas flow	17 L/min
Capillary voltage	Positive 1,500 V Negative 3000V
Nozzle voltage	Positive 300 V Negative 400V

Data relevant to quantification are provided in Table S8B through S8E.

Table S8B the range of concentrations of authentic chemicals used in the analyses.

Table S8C MS parameters and retention times

Table S8D Standard curve results

Table S8E Minimum detection limits

Table S8B. Concentration ranges utilized. (ISTD = internal standard)

Group	Compound	Level1	Level2	Level3	Level4	Level5	Level6	Level7	Unit
Amino Acids	L-Phenylalanine	0.1	0.2	0.5	1	2	5	10	μM/L
Amino Acids	L-Tryptophan	10	20	50	100	200	500	1000	μg/L
Amino Acids	L-Isoleucine	0.1	0.2	0.5	1	2	5	10	μM/L
Amino Acids	L-Leucine	0.1	0.2	0.5	1	2	5	10	μM/L
Amino Acids	L-Methionine	0.1	0.2	0.5	1	2	5	10	μM/L
Amino Acids	L-Asparagine	10	20	50	100	200	500	1000	μg/L
Amino Acids	L-Tyrosine	0.1	0.2	0.5	1	2	5	10	μM/L
Amino Acids	L-Proline	0.1	0.2	0.5	1	2	5	10	μM/L
Amino Acids	L-Valine	0.1	0.2	0.5	1	2	5	10	μM/L
Amino Acids	L-Alanine	0.1	0.2	0.5	1	2	5	10	μM/L
Amino Acids	L-Threonine	0.1	0.2	0.5	1	2	5	10	μM/L
Amino Acids	Glycine	0.1	0.2	0.5	1	2	5	10	μM/L
Amino Acids	L-Glutamic acid	0.1	0.2	0.5	1	2	5	10	μM/L
Amino Acids	L-Aspartic acid	0.1	0.2	0.5	1	2	5	10	μM/L
Amino Acids	L-Serine	0.1	0.2	0.5	1	2	5	10	μM/L
Amino Acids	L-Cystine	0.05	0.1	0.25	0.5	1	2.5	5	μM/L
Amino Acids	L-Histidine	0.1	0.2	0.5	1	2	5	10	μM/L
Amino Acids	L-Lysine	0.1	0.2	0.5	1	2	5	10	μM/L
Amino Acids	L-Arginine	0.1	0.2	0.5	1	2	5	10	μM/L
Amino Acids	L-Cysteine	10	20	50	100	200	500	1000	μg/L
Other	Betaine	10	20	50	100	200	500	1000	μg/L
Siderophore	DMA	0.05	0.1	0.25	0.5	1	2.5	5	μM/L
Other	Syringic acid	10	20	50	100	200	500	1000	μg/L
Other	Coumaric acid	10	20	50	100	200	500	1000	μg/L
Other	Jasmonic Acid	10	20	50	100	200	500	1000	μg/L
Other	Abscisic Acid	10	20	50	100	200	500	1000	μg/L
Other	Ferulic acid	10	20	50	100	200	500	1000	μg/L
Other	Vanillic Acid	10	20	50	100	200	500	1000	μg/L
Other	Salicylic acid	10	20	50	100	200	500	1000	μg/L
Other	Caffeic acid	10	20	50	100	200	500	1000	μg/L
Other	Nicotinic acid	10	20	50	100	200	500	1000	μg/L
Other	Gallic acid	10	20	50	100	200	500	1000	μg/L
ISTD	IS-Ala	1	1	1	1	1	1	1	μM/L
ISTD	IS-Arg	1	1	1	1	1	1	1	μM/L
ISTD	IS-Asn	1	1	1	1	1	1	1	μM/L
ISTD	IS-Asp	1	1	1	1	1	1	1	μM/L

Group	Compound	Level1	Level2	Level3	Level4	Level5	Level6	Level7	Unit
ISTD	IS-Cys	1	1	1	1	1	1	1	µM/L
ISTD	IS-Gln	1	1	1	1	1	1	1	µM/L
ISTD	IS-Glu	1	1	1	1	1	1	1	µM/L
ISTD	IS-Gly	1	1	1	1	1	1	1	µM/L
ISTD	IS-His	1	1	1	1	1	1	1	µM/L
ISTD	IS-Ile	1	1	1	1	1	1	1	µM/L
ISTD	IS-Leu	1	1	1	1	1	1	1	µM/L
ISTD	IS-Lys	1	1	1	1	1	1	1	µM/L
ISTD	IS-Met	1	1	1	1	1	1	1	µM/L
ISTD	IS-Phe	1	1	1	1	1	1	1	µM/L
ISTD	IS-Pro	1	1	1	1	1	1	1	µM/L
ISTD	IS-Ser	1	1	1	1	1	1	1	µM/L
ISTD	IS-Thr	1	1	1	1	1	1	1	µM/L
ISTD	IS-Trp	1	1	1	1	1	1	1	µM/L
ISTD	IS-Tyr	1	1	1	1	1	1	1	µM/L
ISTD	IS-Val	1	1	1	1	1	1	1	µM/L
Organic acids	Gluconate	0.5	1	5	10	15	20	N/A	mg/L
Organic acids	Acetate	0.5	1	5	10	15	20	N/A	mg/L
Organic acids	Formate	0.5	1	5	10	15	20	N/A	mg/L
Organic acids	Malate	0.5	1	5	10	15	20	N/A	mg/L
Organic acids	Citrate	0.5	1	5	10	15	20	N/A	mg/L
Organic acids	Lactate	0.5	1	5	10	15	20	N/A	mg/L
Organic acids	Propionate	0.5	1	5	10	15	20	N/A	mg/L
Organic acids	Butyrate	0.5	1	5	10	15	20	N/A	mg/L
Organic acids	Oxalate	0.5	1	5	10	15	20	N/A	mg/L
Organic acids	2-Oxoglutarate	0.5	1	5	10	15	20	N/A	mg/L
Organic acids	Valerate	0.5	1	5	10	15	20	N/A	mg/L
Organic acids	Pyruvate	0.5	1	5	10	15	20	N/A	mg/L

Table S8C. MS parameters and RT (in minutes)

Group	Compound	RT	Quant Transition	Qual Transition	ISTD Name	ISTD Transition	ISTD RT	Column
Amino Acids	L-Phenylalanine	2.398	166.1 -> 120.1	166.1 -> 103.0	IS-Phe	176.1 -> 129.2	2.406	Intrada AA
Amino Acids	L-Tryptophan	2.553	205.1 -> 188.0	205.1 -> 146.0	IS-Trp	218.1 -> 156.2	2.545	Intrada AA
Amino Acids	L-Isoleucine	2.676	132.1 -> 86.1	132.1 -> 44.2	IS-Ile	139.1 -> 74.1	2.935	Intrada AA
Amino Acids	L-Leucine	2.911	132.1 -> 30.2	132.1 -> 44.2	IS-Leu	139.1 -> 46.2	2.676	Intrada AA
Amino Acids	L-Methionine	3.144	150.1 -> 104.0	150.1 -> 56.1	IS-Met	156.1 -> 109.1	3.143	Intrada AA
Amino Acids	L-Asparagine	3.151	133.1 -> 87.1	133.1 -> 74.0	IS-Asn	139.1 -> 77.1	3.151	Intrada AA
Amino Acids	L-Tyrosine	3.407	182.1 -> 136.1	182.1 -> 91.1	IS-Thr	125.1 -> 78.0	4.714	Intrada AA
Amino Acids	L-Proline	3.464	116.1 -> 70.1	116.1 -> 43.2	IS-Pro	122.0 -> 75.2	3.455	Intrada AA
Amino Acids	L-Valine	3.619	118.1 -> 72.1	118.1 -> 55.1	IS-Val	124.0 -> 77	3.619	Intrada AA
Amino Acids	L-Alanine	4.645	90.1 -> 44.2	90.1 -> 45.3	IS-Ala	93.8 -> 47.2	4.645	Intrada AA
Amino Acids	L-Threonine	4.707	120.1 -> 74.1	120.1 -> 56.1	IS-Thr	125.1 -> 78.0	4.714	Intrada AA
Amino Acids	Glycine	4.826	76.0 -> 43.7	76.0 -> 30.3	IS-Gly	78.8 -> 78.8	4.826	Intrada AA
Amino Acids	L-Glutamic acid	4.895	148.1 -> 84.1	146.0 -> 128.0	IS-Glu	154 -> 89	4.895	Intrada AA
Amino Acids	L-Aspartic acid	5.006	134.0 -> 88.1	134.0 -> 74.0	IS-Asp	139.1 -> 77.1	5.068	Intrada AA
Amino Acids	L-Serine	5.014	106.1 -> 42.2	106.1 -> 60.0	IS-Ser	109.9 -> 63.1	5.03	Intrada AA
Amino Acids	L-Cystine	5.967	122.0 -> 59.1	122.0 -> 76.0	IS-Thr	125.1 -> 78.0	4.714	Intrada AA
Amino Acids	L-Histidine	7.812	156.1 -> 110.1	156.1 -> 83.1	IS-His	165.1 -> 118.0	7.812	Intrada AA
Amino Acids	L-Lysine	8.363	147.1 -> 84.1	147.1 -> 130.1	IS-Lys	155.1 -> 90.0	8.368	Intrada AA
Amino Acids	L-Arginine	8.906	175.1 -> 70.1	175.1 -> 60.1	IS-Arg	185.1 -> 75.1	8.911	Intrada AA
Amino Acids	L-Cysteine	4.1	122 -> 75	122 -> 59.1	IS-Thr	125.1 -> 78.0	4.714	Intrada AA
Amino Acids	Glutamine	5.2	147.1 -> 130	147.1 -> 84	IS-Glu	154 -> 89	4.895	Intrada AA
Other	Betaine	2.51	118.1 -> 58.0	257.1 -> 95.0	IS-Phe	176.1 -> 129.2	2.406	Intrada AA
Siderophore	DMA	1.1	305.1 -> 186.0	305.1 -> 287.0	IS-Trp	125.1 -> 78.0	4.28	C18
Other	Syringic acid	0.9	197.0 -> 151.0	197.0 -> 182.0	IS-Phe	176.1 -> 129.2	2.406	Intrada AA
Other	Coumaric acid	1.332	165.0 -> 147.0	165.0 -> 119.0	IS-Trp	156.1 -> 109.1	4.28	C18
Other	Jasmonic Acid	6.855	209.1 -> 59.0	209.1 -> 165.1	IS-Phe	176.1 -> 129.2	2.882	C18
Other	Abscisic Acid	6.277	265.1 -> 247.1	265.1 -> 187.1	IS-Phe	176.1 -> 129.2	2.882	C18
Other	Ferulic acid	0.5	193.0 -> 178.0	193.0 -> 134.0	IS-Phe	176.1 -> 129.2	2.406	Intrada AA
Other	Vanillic Acid	4.86	166.9 -> 108.0	166.9 -> 137.0	IS-Phe	176.1 -> 129.2	2.882	C18
Other	Salicylic acid	1.1	137.0 -> 93.1	137.0 -> 65.2	IS-Phe	176.1 -> 129.2	2.406	Intrada AA
Other	Caffeic acid	6.831	178.9 -> 135.0	178.9 -> 89.0	IS-Phe	176.1 -> 129.2	2.882	C18
Other	Nicotinic acid	1.05	124.0 -> 80.0	122.0 -> 51.2	IS-Phe	176.1 -> 129.2	2.882	C18
Other	Gallic acid	1.1	168.9 -> 124.9	168.9 -> 79.1	IS-Phe	176.1 -> 129.2	2.406	Intrada AA
Other	Trehalose	2	365.0 -> 203.0	341.0 -> 59.0	IS-Phe	176.1 -> 129.2	2.406	Intrada AA

Group	Compound	RT	Quant Transition	Qual Transition	ISTD Name	ISTD Transition	ISTD RT	Column
Other	Spermine	4.5	203.2 -> 209.1	203.2 -> 112.1	IS-Thr	125.1 -> 78.0	4.714	Intrada AA
Other	Spermidine	4.5	146.2 -> 72.1	146.2 -> 129.1	IS-Thr	125.1 -> 78.0	4.714	Intrada AA
Other	Putrescine	0.703	89.0 -> 30.0	89.0 -> 72.0	IS-Trp	125.1 -> 78.0	4.28	C18
Organic acids	Gluconate	8.573	N/A	N/A	N/A	N/A	N/A	ThermoFisher AS11HC
Organic acids	Acetate	9.780	N/A	N/A	N/A	N/A	N/A	ThermoFisher AS11HC
Organic acids	Formate	12.110	N/A	N/A	N/A	N/A	N/A	ThermoFisher AS11HC
Organic acids	Malate	25.290	N/A	N/A	N/A	N/A	N/A	ThermoFisher AS11HC
Organic acids	Citrate	37.970	N/A	N/A	N/A	N/A	N/A	ThermoFisher AS11HC
Organic acids	Lactate	9.07	N/A	N/A	N/A	N/A	N/A	ThermoFisher AS11HC
Organic acids	Propionate	11.320	N/A	N/A	N/A	N/A	N/A	ThermoFisher AS11HC
Organic acids	Butyrate	12.673	N/A	N/A	N/A	N/A	N/A	ThermoFisher AS11HC
Organic acids	Oxalate	29.243	N/A	N/A	N/A	N/A	N/A	ThermoFisher AS11HC
Organic acids	2-Oxoglutarate	27.917	N/A	N/A	N/A	N/A	N/A	ThermoFisher AS11HC
Organic acids	Valerate	15.113	N/A	N/A	N/A	N/A	N/A	ThermoFisher AS11HC
Organic acids	Pyruvate	13.297	N/A	N/A	N/A	N/A	N/A	ThermoFisher AS11HC

The correlation coefficient R^2 of all reported compounds is not less than 0.99 as demonstrated by the data in Table S8D.

Table S8D. Standard curve results

Group	Compound	Curve Fit	Curve Fit Formula	Curve Fit R^2
Amino Acids	L-Phenylalanine	Linear	y = 9.011543 * x - 0.047623	0.9998
Amino Acids	L-Tryptophan	Linear	y = 11.842789 * x - 0.091137	0.9992
Amino Acids	L-Isoleucine	Linear	y = 54.567262 * x - 0.419924	0.9992
Amino Acids	L-Leucine	Linear	y = 5.362601 * x - 0.030544	0.9974
Amino Acids	L-Methionine	Linear	y = 20.001171 * x - 0.112620	0.9989
Amino Acids	L-Asparagine	Linear	y = 6.303982 * x + 0.060418	0.9982
Amino Acids	L-Tyrosine	Linear	y = 7.878483 * x - 0.112140	0.9969
Amino Acids	L-Proline	Linear	y = 4.798481 * x - 0.029112	0.9996
Amino Acids	L-Valine	Linear	y = 5.332735 * x - 0.039520	0.9991
Amino Acids	L-Alanine	Linear	y = 3.470595 * x - 0.024634	0.9990
Amino Acids	L-Threonine	Linear	y = 6.557110 * x + 0.032257	0.9988
Amino Acids	Glycine	Linear	y = 3.816395 * x - 0.096900	0.9842
Amino Acids	L-Glutamic acid	Linear	y = 1.882982 * x + 0.006850	0.9998
Amino Acids	L-Aspartic acid	Linear	y = 0.474376 * x + 0.009206	0.9993
Amino Acids	L-Serine	Linear	y = 2.162862 * x + 0.159566	0.9985
Amino Acids	L-Cystine	Linear	y = 3.667328 * x + 0.033396	0.9990
Amino Acids	L-Histidine	Linear	y = 29.175062 * x + 0.253324	0.9960
Amino Acids	L-Lysine	Linear	y = 5.958616 * x + 0.104629	0.9980
Amino Acids	L-Arginine	Linear	y = 1.227602 * x + 0.001103	0.9991
Amino Acids	L-Cysteine	Linear		
Amino acids	Glutamine	Linear	y = 1.299854 * x - 0.012777	0.9986
Other	Betaine	Linear	y = 193.344141 * x - 1.014497	0.9999
Siderophores	DMA	Linear	y = 0.048859 * x - 0.001887	0.9940
Other	Syringic acid	Linear	y = 0.119196 * x + 0.094255	0.9401
Other	Coumaric acid	Linear	y = 0.008448 * x - 1.559670E-004	0.9930
Other	Jasmonic Acid	Linear	y = 0.264706 * x - 0.008222	0.9942
Other	Abscisic Acid	Linear	y = 0.035792 * x - 6.597420E-004	0.9923

Group	Compound	Curve Fit	Curve Fit Formula	Curve Fit R^2
Other	Ferulic acid	Linear	y = 0.027037 * x - 1.510547E-005	0.9972
Other	Vanillic Acid	Linear	y = 0.023836 * x - 4.167909E-004	0.9974
Other	Salicylic acid	Linear	y = 0.944205 * x + 0.013260	0.9985
Other	Caffeic acid	Linear	y = 0.183273 * x - 0.001184	0.9935
Other	Nicotinic acid	Linear	y = 0.878263 * x - 0.021481	0.9967
Other	Gallic acid	Linear	y = 0.140877 * x - 0.001663	0.9972
Other	Trehalose	Linear	y = 15.020935 * x + 12.720002	0.9772
Other	Spermine	Linear	y = 0.007128 * x + 0.001263	0.9904
Other	Spermidine	Linear	y = 4.455273 * x - 0.155584	0.9845
Other	Putrescine	Linear	y = 0.948373 * x + 0.021457	0.9913
Organic acids	Gluconate	Linear	Y = 0.0411 * x	0.999
Organic acids	Acetate	Linear	Y = 0.1191 * x	0.999
Organic acids	Formate	Linear	Y = 0.1865 * x	0.999
Organic acids	Malate	Linear	Y = 0.0995 * x	0.999
Organic acids	Citrate	Linear	Y = 0.0566 * x	0.999
Organic acids	Lactate	Linear	Y = 0.0892 * x	0.999
Organic acids	Propionate	Linear	Y = 0.0861 * x	0.999
Organic acids	Butyrate	Linear	Y = 0.0773 * x	0.999
Organic acids	Oxalate	Linear	Y = 0.3512 * x	0.999
Organic acids	2-Oxoglutarate	Linear	Y = 0.0850 * x	0.999
Organic acids	Valerate	Linear	Y = 0.0677 * x	0.999
Organic acids	Pyruvate	Linear	Y = 0.0820 * x	0.999

The minimum detection limit (MDL) or lowest detection limit is determined from seven injections of the lowest concentration and evaluated using the SW846 - evaluation method, 3 times the standard deviation.

Table S8E. Minimum detection limits

Group	Compound	MDL Avg Conc.	LOD	LOQ	Noise	S/N	Unit
Amino Acids	L-Phenylalanine	0.144	0.019	0.065	20.36	55.94	μM/L
Amino Acids	L-Tryptophan	13.1	2	6.8	50.69	18.08	μg/L
Amino Acids	L-Isoleucine	0.097	0.067	0.224	12.53	8.52	μM/L
Amino Acids	L-Leucine	0.1	0.025	0.084	13.36	2.36	μM/L
Amino Acids	L-Methionine	0.138	0.039	0.131	8.65	21.72	μM/L
Amino Acids	L-Asparagine	0.164	0.033	0.111	6.48	∞	μM/L
Amino Acids	L-Tyrosine	0.071	0.021	0.069	17.81	20.73	μM/L
Amino Acids	L-Proline	0.108	0.012	0.04	25.16	∞	μM/L
Amino Acids	L-Valine	0.183	0.114	0.381	19.83	1.43	μM/L
Amino Acids	L-Alanine	0.272	0.402	1.341	44.74	0.41	μM/L
Amino Acids	L-Threonine	0.103	0.082	0.275	14.32	2.25	μM/L
Amino Acids	Glycine	2.77	2.298	7.659	29.15	1.77	μM/L
Amino Acids	L-Glutamic acid	0.092	0.069	0.231	9.61	23.11	μM/L
Amino Acids	L-Aspartic acid	0.094	0.07	0.234	14.5	12.86	μM/L
Amino Acids	L-Serine	1.211	2.796	9.318	29.37	0.97	μM/L
Amino Acids	L-Cystine	0.07	0.089	0.295	0.3	34.92	μM/L
Amino Acids	L-Histidine	0.136	0.023	0.076	21.46	218.91	μM/L
Amino Acids	L-Lysine	0.115	0.078	0.261	4.9	10.49	μM/L
Amino Acids	L-Arginine	0.126	0.012	0.039	19.74	241.67	μM/L
Amino Acids	L-Cysteine	6.19	2.81	9.36	8.25	20.77	μg/L
Amino Acids	Glutamine	10	0.51	1.77	-	59.2	μg/L
Other	Betaine	13.7	1.5	5	1000.69	43.88	μg/L
Siderophore	DMA	0.076	0.048	0.161	0.3	378.8	μM/L
Other	Syringic acid	20.7	16.3	54.3	0.29	107.82	μg/L
Other	Coumaric acid	18	12.7	42.3	21.6	4.2	μg/L
Other	Jasmonic Acid	15.7	3.3	10.9	0.06	∞	μg/L
Other	Abscisic Acid	32	18.1	60.4	34.85	6.55	μg/L
Other	Ferulic acid	25.9	19.9	66.2	0.19	∞	μg/L
Other	Vanillic Acid	33.8	28.1	93.7	0.24	32.16	μg/L
Other	Salicylic acid	26.3	5.5	18.2	9.9	∞	μg/L
Other	Caffeic acid	18.7	6.3	20.9	4.9	∞	μg/L

Group	Compound	MDL Avg Conc.	LOD	LOQ	Noise	S/N	Unit
Other	Nicotinic acid	10.1	3.6	12.1	6.43	∞	µg/L
Other	Gallic acid	25	22.8	75.9	7.53	6.45	µg/L
Organic acids	Gluconate	0.03	N/A	N/A	N/A	N/A	mg/L
Organic acids	Acetate	0.05	N/A	N/A	N/A	N/A	mg/L
Organic acids	Formate	0.01	N/A	N/A	N/A	N/A	mg/L
Organic acids	Malate	0.09	N/A	N/A	N/A	N/A	mg/L
Organic acids	Citrate	0.03	N/A	N/A	N/A	N/A	mg/L
Organic acids	Lactate	0.09	N/A	N/A	N/A	N/A	mg/L
Organic acids	Propionate	0.03	N/A	N/A	N/A	N/A	mg/L
Organic acids	Butyrate	0.03	N/A	N/A	N/A	N/A	mg/L
Organic acids	Oxalate	0.0	N/A	N/A	N/A	N/A	mg/L
Organic acids	2-Oxoglutarate	0.09	N/A	N/A	N/A	N/A	mg/L
Organic acids	Valerate	0.02	N/A	N/A	N/A	N/A	mg/L
Organic acids	Pyruvate	0.09	N/A	N/A	N/A	N/A	mg/L

Supplemental Figure S1. Microbial colony growth from root washes.

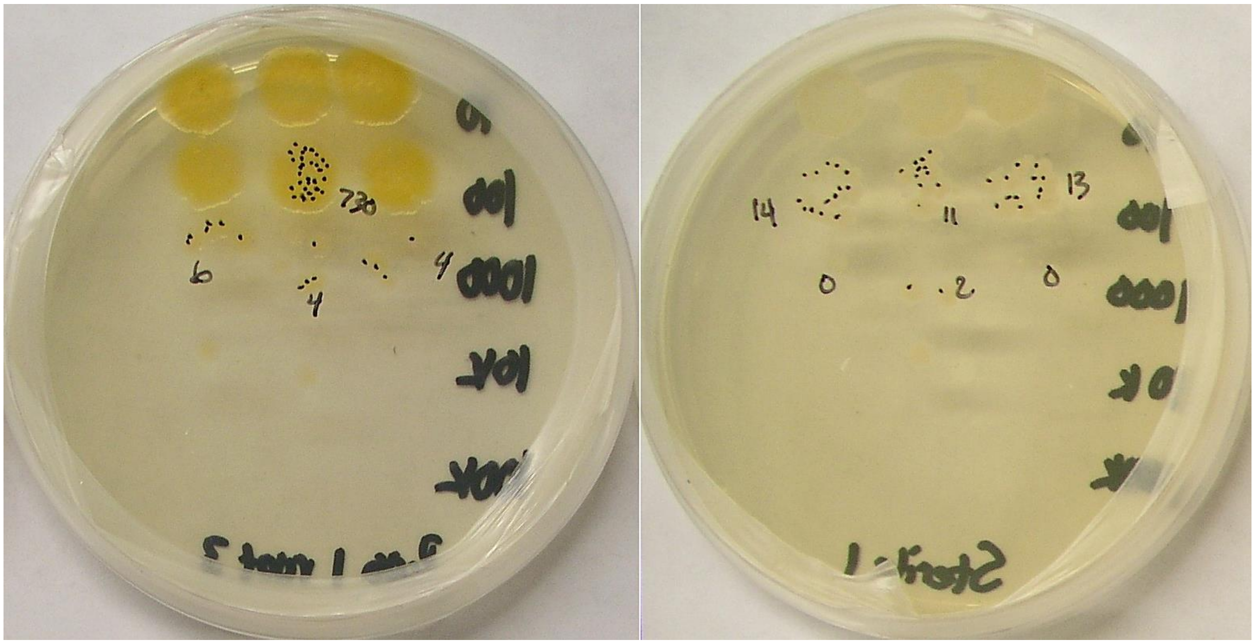

Images of typical agar plates showing (Left) colonies of *Pc*O6 as obtained from dilution plating of root washes from *Pc*O6 - inoculated plants, or (Right) the white colonies obtained from the root washes only of the noninoculated plants grown under drought stress. These plates are examples of those used to determine cfu/cm root at harvest as reported in the text.

Supplemental Figure S2A,B. These figures show the anticipated major metabolic pathways in wheat. They depict how low molecular weight organic acid metabolites relate to the TCA cycle (A) and to the synthesis of amino acid synthesis (B). Data are from: [93-94].

The blue arrows in Figure A show the shunt that replaces part of the TCA cycle yet allows levels of malate to be maintained.

Supplemental Figure S2A

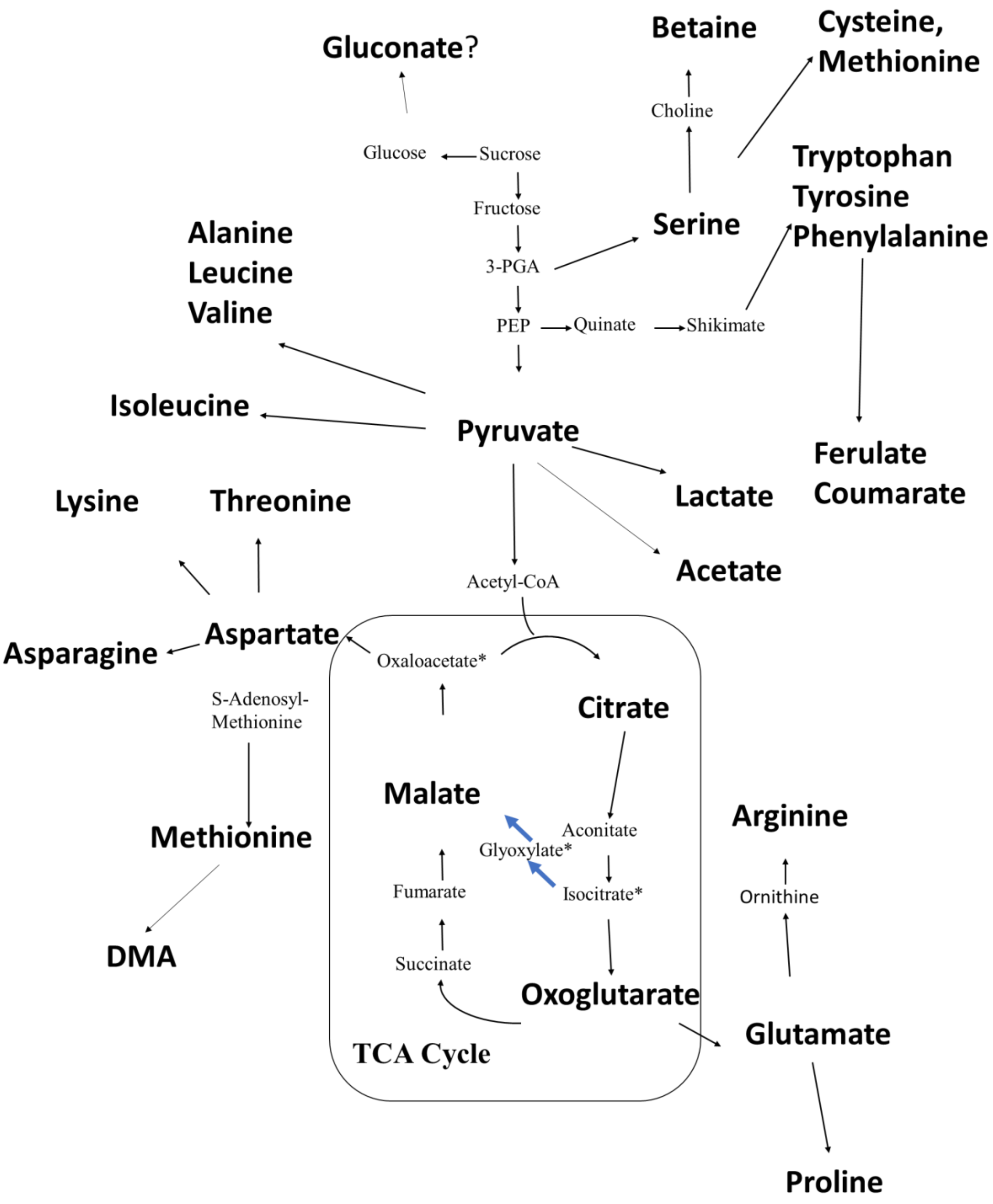

Supplemental Figure S2B.

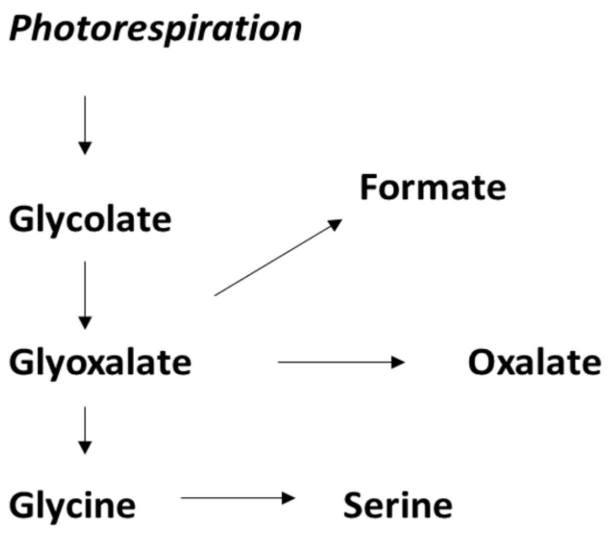

Supplemental Figure S3. Quality control studies for shoot extracts.

Some metabolites in the shoot extracts did not pass quality control when tested. Thus, these metabolites although detected in some extracts were not discussed in the text of the paper. The metabolites were caffeic acid, GABA, glutamine, nicotinic acid and trehalose. Other metabolites had better consistency in recoveries but still sometimes fell outside 75-125% recovery. For these metabolites, laboratory control sample recovery and matrix extraction efficiencies are shown in Figure S4 with n = 4 for laboratory control samples (analyte spiked into DI water and extracted) and n = 14 for matrix extraction (analyte spiked into extracted solution just prior to analysis) efficiencies. Averages and standard deviations are shown. The beige shaded portion shows the zone of 75-125% recovery.

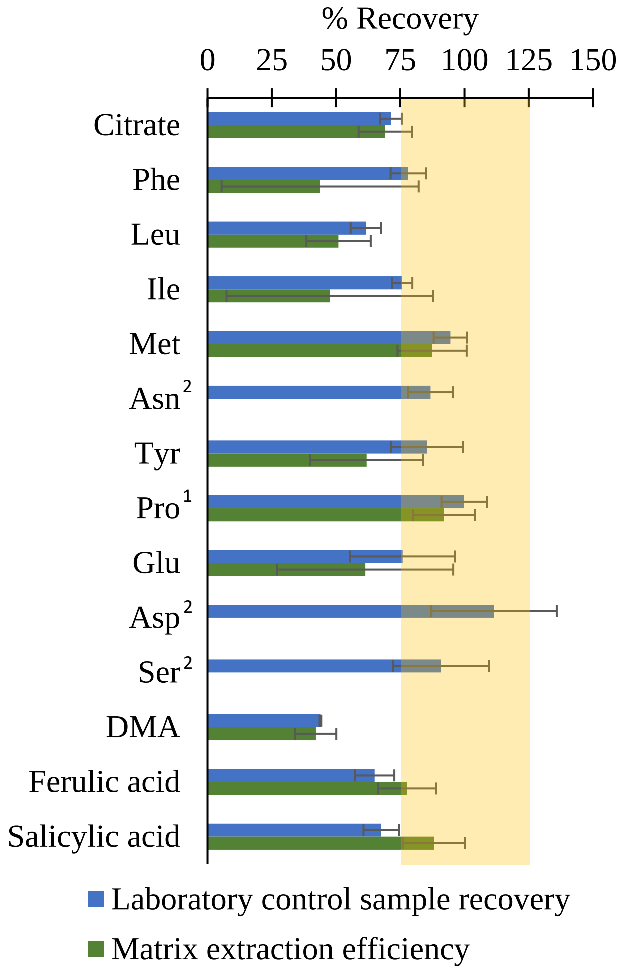

[1]For proline, only the matrix extraction efficiencies of the well-watered treatments (n = 6) are shown; the matrix extraction efficiencies of the droughted treatments (n = 8) were < 0% due to the high background concentrations.
[2]For these compounds, matrix extraction efficiencies are not reported despite acceptable laboratory control sample recoveries; the matrix extraction efficiencies were < 0% due to the high background concentrations.
